# NorWood: a gene expression resource for evo‐devo studies of conifer wood development

**DOI:** 10.1111/nph.14458

**Published:** 2017-02-10

**Authors:** Soile Jokipii‐Lukkari, David Sundell, Ove Nilsson, Torgeir R. Hvidsten, Nathaniel R. Street, Hannele Tuominen

**Affiliations:** ^1^ Umeå Plant Science Centre Department of Plant Physiology Umeå University SE‐901 87 Umeå Sweden; ^2^ Umeå Plant Science Centre Department of Forest Genetics and Plant Physiology Swedish University of Agricultural Sciences SE‐901 84 Umeå Sweden; ^3^ Department of Chemistry, Biotechnology and Food Science Norwegian University of Life Sciences 1430 Ås Norway

**Keywords:** co‐expression network, comparative genomics, cryosection, evo‐devo, growth ring, Norway spruce (*Picea abies*), RNA‐sequencing, secondary cell wall

## Abstract

- The secondary xylem of conifers is composed mainly of tracheids that differ anatomically and chemically from angiosperm xylem cells. There is currently no high‐spatial‐resolution data available profiling gene expression during wood formation for any coniferous species, which limits insight into tracheid development.
- RNA‐sequencing data from replicated, high‐spatial‐resolution section series throughout the cambial and woody tissues of *Picea abies* were used to generate the NorWood.conGenIE.org web resource, which facilitates exploration of the associated gene expression profiles and co‐expression networks.
- Integration within PlantGenIE.org enabled a comparative regulomics analysis, revealing divergent co‐expression networks between *P. abies* and the two angiosperm species *Arabidopsis thaliana* and *Populus tremula* for the secondary cell wall (SCW) master regulator *NAC Class IIB* transcription factors. The SCW *cellulose synthase* genes (*CesA*s) were located in the neighbourhoods of the *NAC* factors in *A*. *thaliana* and *P. tremula*, but not in *P*. *abies*. The NorWood co‐expression network enabled identification of potential SCW *CesA* regulators in *P. abies*.
- The NorWood web resource represents a powerful community tool for generating evo‐devo insights into the divergence of wood formation between angiosperms and gymnosperms and for advancing understanding of the regulation of wood development in *P. abies*.

## Introduction

Conifers are woody plants representing the largest lineage of the ancient seed plant group of gymnosperms (Wang & Ran, 2014). Despite the relatively small number of extant species, conifers dominate vast areas, especially in boreal forests of the northern hemisphere, playing an import role in carbon cycling and ecosystem function. Conifers are important producers of raw material for paper, solid fuels, liquid biofuels and biomaterials (Guo *et al*., 2015; Isikgor & Becer, 2015).

The majority of conifer species are large, slow‐growing trees. They share several properties with angiosperm trees, such as the formation of extensive secondary xylem (the ‘wood’), which facilitates water and nutrient transport in addition to mechanical support. However, there are striking anatomical differences in how these functions are achieved: while conifer wood consists mainly of single‐celled tracheids that serve both water transport and physical support functions, angiosperm wood is composed of water‐transporting multicellular conduits called vessels and libriform fibres, which provide structural support.

Secondary cell wall (SCW) properties are one of the primary determinants of xylem element function. The SCW is deposited inside the primary cell wall and is composed of polysaccharidic cellulose and hemicellulose and the polyphenolic compound lignin. Current knowledge of SCW formation derives largely from studies of *Arabidopsis thaliana*, which have identified the importance of NAC (NAM, ATAF1/2 and CUC2) domain proteins in the regulation of SCW formation. NAC SECONDARY WALL THICKENING PROMOTING FACTOR1 (NST1) and NST2 function redundantly in regulating SCW thickening in the endothecium of anthers (Mitsuda *et al*., 2005), whereas NST1 and NST3 (also known as SND1, SECONDARY WALL‐ASSOCIATED NAC DOMAIN PROTEIN1) regulate SCW thickening of xylem fibres (Mitsuda *et al*., 2007; Zhong *et al*., 2007). VASCULAR‐RELATED NAC‐DOMAIN6 (VND6) and VND7, on the other hand, control formation of vessel elements (VNDs; Kubo *et al*., 2005; Yamaguchi *et al*., 2008). These SCW ‘master switches’ regulate transcription of downstream transcription factors (TFs), including MYB (myeloblastosis) family proteins, which further regulate other downstream TFs and, finally, the structural SCW biosynthetic genes (Hussey *et al*., 2013), including SCW *cellulose synthase* genes (*CesAs*). Many of the upstream TFs regulate both their downstream TFs and the cell wall biosynthetic genes directly (feed‐forward regulation), indicating the complexity of the regulatory network (Hussey *et al*., 2013; Taylor‐Teeples *et al*., 2015).

The anatomical differences between gymnosperm and angiosperm xylem elements suggest corresponding differences in the regulation of xylem formation and differentiation. A few studies have been performed to elucidate these aspects in conifer trees. R2R3 MYB TF family members, which are implicated in the biosynthesis of phenylpropanoids and flavonoids, display conserved DNA binding domains between angiosperms and gymnosperms (Bedon *et al*., 2007), and evidence for functional conservation has been reported for two R2R3 MYB factors in lignin biosynthesis (Bomal *et al*., 2008). Duval *et al*. (2014) performed a protein–DNA interaction study in *Picea glauca* for a number of xylem‐expressed TFs, identifying regulatory networks that were broadly similar to those in *A. thaliana*. Functional conservation of the master switch NAC TFs in the Class IIB family (also referred to as WNDs (Wood‐Associated NAC Domain), VNS (VND/NST/SMB) or SWNs (Secondary Wall NACs)) has not been investigated, although homologous members of the family were reported in *Picea abies* (Nystedt *et al*., 2013).

Detailed understanding of the regulatory aspects of wood development requires analyses of gene expression with high spatial resolution. This is not easily achievable in *A. thaliana* due to the small size of plants and the minor extent of secondary xylem formation. To overcome these limitations, angiosperm trees including members of the genus *Populus* and their hybrids have emerged as powerful alternative models. Several gene expression studies have been performed using cryogenic tangential cutting series (hereafter cryo‐series) assayed with cDNA gene expression microarrays (Hertzberg *et al*., 2001; Schrader *et al*., 2004; Moreau *et al*., 2005; Courtois‐Moreau *et al*., 2009) or RNA‐sequencing (RNA‐seq; Immanen *et al*., 2016). These provided the first insights into detailed patterns of gene expression across the vascular cambium, differentiating secondary phloem and xylem within a single annual growth ring. In conifers, global gene expression studies on different wood types, such as earlywood vs latewood (Paiva *et al*., 2008; Li *et al*., 2010; Raherison *et al*., 2015), compression wood vs normal wood (Villalobos *et al*., 2012) and ‘high density’ wood vs ‘low density’ wood (Stephenson *et al*., 2011), have provided views of the co‐expression networks active during xylem development. However, these were limited either by restricted coverage of the transcriptome, specificity or dynamic range (in the case of cDNA arrays) and/or by limitations in sampling resolution.

The availability of draft conifer genome assemblies (De La Torre *et al*., 2014) affords new opportunities for evolutionary, evo‐devo and comparative genomics studies. Here, the *P*. *abies* genome (Nystedt *et al*., 2013) was used together with a novel high‐spatial‐resolution RNA‐seq gene expression resource to profile the developmental process of secondary xylem formation in mature *P*. *abies* trees growing under natural conditions. A web resource ‘NorWood’ (http://NorWood.ConGenIE.org) was implemented for data exploration, visualisation and integration with the PlantGenIE.org platform (Sundell *et al*., 2015). The potential of the NorWood resource is exemplified through comparative analyses of co‐expression networks in *A. thaliana*,* Populus* and *P. abies*, demonstrating that the resource captures existing knowledge and provides novel insight into the evolutionary divergence of xylem differentiation.

## Materials and Methods

### Plant material

Samples were collected from 49‐yr‐old Norway spruce (*P. abies* [L.] Karst.) trees growing at a seed orchard in Hissjö (Västerbotten, Sweden). The sampled trees represented clonal copies of genotype ‘Z4006’ (see Supporting Information Notes S1, section 1.1), the genotype used for the *P*. *abies* genome sequence (Nystedt *et al*., 2013). Wood pieces were collected from three replicate trees at the end of July 2012 (see ‘Collection of stem samples’) when cell death of newly formed earlywood was ongoing but before latewood cells could be observed based on microscopic examination (see below). All samples were collected at noon.

### Collection of stem samples with intact cambium

Samples were taken at breast height in a part of the stem free of branches or traumatic resin ducts. To preserve intact cambium the bark, phloem and part of the wood was removed from a *c*. 3 cm × 10 cm region around the sampling area using a carpet knife. This released the shearing forces present in the stem. Samples covering bark, phloem, cambium, developing xylem and two to three previous year rings were then taken by hammering a chisel into the stem at a 90° angle immediately above the pieces and hammering tangentially downwards. The samples used for RNA‐seq were immediately frozen in liquid N_2_ and kept on dry ice until storage at −80°C. Stem samples used for light microscopy were kept on ice until preparation for microscopy.

### Light microscopy of fresh tissues

Cell viability and the location of the cell death zone were determined by staining the fresh tissues with nitroblue tetrazolium (NBT; Berlyn & Miksche, 1976; Gahan, 1984), which in the presence of succinate indicates succinate dehydrogenase activity of living cells. Cross‐sections of *c*. 30 μm were cut using a cylinder hand microtome and incubated for 1.5 h in 50 mM sodium phosphate buffer (pH 7.6) containing 50 mM sodium succinate and 500 mg l^−1^ NBT under strong light conditions. Sections were examined using an Axioplan 2 microscope (Zeiss) and micrographs captured with an AxioCam HRC camera (Zeiss). The abundance of living xylem cells was evaluated by examining the captured images using the axiovision LE software (Rel. 4.9.1; Zeiss). Data were collected from three separate sections per tree and at three positions in each section (left, middle and right).

### Collecting tangential cryogenic sections

To assay gene expression at high spatial resolution across the vascular development zone, a continuous cryo‐section series was taken starting in the functional phloem and ending in the latewood from the previous year. A band saw was used to rapidly trim the frozen blocks, after which specimens were mounted into OCT media (VWR, Radnor, PA, USA) at −25°C and orientated parallel to the cambium according to Uggla *et al*. (1996). Tangential 30 μm sections, the average tracheid diameter in *P*. *abies* (Havimo *et al*., 2008), and *c*. 5 × 25 mm in size were sectioned using a steel blade in an HM 505 E microtome (Microm Laborgeräte, Walldorf, Germany) and stored at −80°C. Transverse sections were taken from the tissue block and used to confirm the composition of tissue types in each tangential section.

### RNA extraction and amplification

Total RNA from the sections was extracted using the miRNeasy Micro Kit (Qiagen) according to the manufacturer's instructions. The number of cryosections collected from each tree ranged from 44 to 82. Sections representing cambium or newly formed xylem were extracted individually, whereas sections from other regions of the series were pooled (Table S1) to increase the amount of isolated RNA and to even out differences among replicate trees in the width of the year ring. These are hereafter referred to as samples, which were numbered in ascending numerical order from ‐01 for the first sample on the phloem side. The replicate trees are referred to as T1–T3.

Homogenisation of the material was performed with metal beads in lysis buffer using a bead mill (25 Hz, 3 min; Retsch, Haan, Germany). Before purification the optional DNase treatment was performed. RNA concentration and purity were measured using a NanoDrop 2000 spectrophotometer (NanoDrop Technologies, Wilmington, DE, USA) and integrity was analysed on an Agilent 2100 Bioanalyzer with Pico chips (Agilent Technologies, Waldbronn, Germany). mRNA was amplified using the MessageAmpII aRNA Amplification Kit (Thermo Fisher Scientific, Waltham, MA, USA) according to the manufacturer's instructions. It was not possible to extract adequate RNA from samples ‐01 and ‐02 from trees T1–T3. As a result, the phloem is not represented in the current data.

### Data processing and quality control

Sequencing of the amplified RNA (aRNA) was conducted at SciLifeLab (Stockholm, Sweden) using an Illumina HiSeq 2500, as in Nystedt *et al*. (2013). Paired‐end reads (2 × 125 bp, target insert size 250 bp) to a minimum sequence depth of 10 million reads per sample were generated. Reads were pre‐processed as in Delhomme *et al*. (2014). Pre‐processing steps comprised FastQC‐0.11.2 (http://www.bioinformatics.babraham.ac.uk/projects/fastqc/) for quality control, SortmeRNA‐2.0 to filter RNA contaminants, Trimmomatic‐0.36 (Bolger *et al*., 2014; ILLUMINACLIP:2:30:10) for adapter removal and read trimming, STAR‐2.0.3 (Dobin *et al*., 2013; –outSAMmapqUnique 254 –quantMode TranscriptomeSAM –outFilterMultimapNmax 100 –chimSegmentMin 1) for read alignment and HTSeq‐0.6.1 (‐m intersection‐nonempty ‐s yes ‐t exon ‐i Parent; http://www-huber.embl.de/users/anders/HTSeq/) to count aligned reads. Read counts were normalised and variance stabilized transformed (VST) to obtain gene expression values using the R‐package DESeq2‐1.12 (Love *et al*., 2014). A filter requiring VST > 3 in two samples per tree in at least two of the three replicate trees was applied, resulting in 18 513 expressed genes (25% of the gene space). One sample (T2‐15) was not sent for sequencing and one sample (T2‐10) had insufficient reads after sequencing. Both were replaced by the average of the samples immediately before and after the missing sample.

### Data availability

Sequencing data are available at the European Nucleotide Archive as accession ERP017340.

### Co‐expression network and network connectivity measures

A co‐expression network was calculated for all 18 513 expressed genes. Mutual information (MI; Daub *et al*., 2004) was used to calculate pairwise correlation between genes and context‐likelihood of relatedness (CLR) to transform these correlations into a background corrected *Z*‐score (Faith *et al*., 2007; Netotea *et al*., 2014). Briefly, MI is a popular nonparametric correlation measure that is robust to outliers, while the CLR algorithm performs a local background correction that serves to effectively remove false positive correlations. The method computes a *Z*‐score for each pair of genes using a null distribution obtained from the scores between these two genes and all other genes. Further discussion of MI/CLR can be found in Netotea *et al*. (2014). A network was then constructed by linking all gene‐pairs with a *Z*‐score above a determined CLR threshold (*Z*‐score ≥ 5). This CLR threshold was determined using a scale freeness test, based on previous analysis showing that biological networks have scale‐free properties (Barabási & Oltvai, 2004; MacNeil & Walhout, 2011). After applying the CLR threshold, 17 942 genes were part of the largest sub‐network, having at least one neighbour, and were thus included in the network. The vast majority of genes not contained in this largest subnetwork were present as disconnected nodes. Since MI measures the dependency between two expression profiles regardless of the sign of that dependency, edges in the network were annotated as positive or negative using Pearson correlation. The network was subsequently used to calculate network centrality scores for each gene: degree centrality, the number of direct neighbours (first‐order neighbours) of a gene in the network; betweenness centrality, the number of shortest paths passing through a gene, where the shortest path is the smallest number of edges connecting a pair of genes; closeness centrality, the minimum total distance from a gene to all other genes; and average neighbour degree, the average number of neighbours of a gene′s direct neighbours. The betweenness and closeness centrality scores were ranked from the highest to lowest to obtain rank order scores. Network centralities were calculated using the python package networkx‐1.9 (https://networkx.github.io/).

### TF annotation, hierarchical clustering and gene ontology enrichment

TF annotations for *P. abies* were obtained from the Plant Transcription Factor Database (PlantTFDB; Jin *et al*., 2016). Unsupervised clustering was performed using the R package gplots‐2_2.1.0 (Warnes *et al*., 2016) and the heatmap2 function from the R package gplots (Warnes *et al*., 2016). The hierarchical dendrograms were calculated using Ward's method, and were based on Euclidian distance for the samples and Pearson correlation for genes. In total, 7788 expressed genes with a variance of σ > 1 across all samples were included in the clustering. After visual examination of the cluster dendrogram, this was cut to define seven gene expression clusters.

In addition to the hierarchical clustering, each gene was assigned to the sample(s) in which the expression of that gene was within 4% of the maximum expression for that gene across all samples. Gene ontology (GO) enrichment was then tested for the set of genes assigned to each sample to annotate the developmental gradient. As the samples did not align exactly across the three replicate cutting series, this analysis was performed using only tree T1.

GO enrichment tests were performed for all hierarchical clusters and sample clusters using Fisher's exact test in the python package fisher‐0.1.4 (https://pypi.python.org/pypi/fisher/) and false discovery rate (FDR) correction.

### NorWood implementation

The Norwood web resource (http://norwood.congenie.org) was implemented using a combination of server and user side scripting languages (HTML‐5, python‐2.7, PhP‐5, jquery‐1.9 and javascript‐1.8). All expression, network and annotation data were stored and managed using a MySQL‐5.7 database. PhP‐mysqli and MySQL‐python‐1.2.5 were used for web management. Gene profiles were plotted using the python matplotlib‐1.5.1 library. Networks were displayed using cytoscapeweb 1.0.4 (Lopes *et al*., 2010) and heatmaps using the heatmap2 function in R. The gene information and functional enrichment tables were implemented using the javascript datatables‐1.9 plugin and CSS design. The web resource layout was designed using HTML‐5 CSS styling. All scripts are available at https://github.com/UPSCb/NorWood.

### Phylogenetic analyses of *VND*,* NST* and *CesA* genes

Blastp searches of *A*. *thaliana NST* and *CesA* genes were performed against the *P*. *abies* genome at ConGenIE.org. The *P. abies*,* A*. *thaliana* and *P*. *trichocarpa* gene family members of the identified sequences were extracted from the associated gene information pages (Gene Family tab). *P*. *abies* genes expressed in the NorWood cryo‐section series, *Populus* *trichocarpa* genes expressed within the RNA‐seq data at PopGenIE.org, and genes that were full‐length compared to *A*. *thaliana* sequences were included in subsequent analyses. Two very short *CesA* superfamily sequences (MA_10429177g0020 and Potri.011G152300) were discarded. In *P*. *abies* some *CesA* gene models were truncated, and further analyses of RNA‐seq read alignments, Trinity transcript assemblies available at ConGenIE.org and *de novo* gene prediction all confirmed two cases where a combination of *CesA* gene fragments could be merged to form a full‐length gene model (Notes S1, section 1.2). These were then used for phylogenetic analyses. For downstream expression analysis, we used the longest of the original fragmented gene models. Phylogenetic trees were created using the galaxy platform (Goecks *et al*., 2010) hosted at PlantGenIE.org. The workflow utilises muscle v.3.8.31 (Edgar, 2004; maximum number of iterations: 16) for multiple alignment, and phyML 3.1 (Guindon *et al*., 2010; substitution model: WAG, aLRT test: SH‐like, tree topology search operation: Nearest Neighbor Interchange) and treevector (Pethica *et al*., 2010) for building and drawing phylogenetic trees, respectively. For the *NAC* gene family tree, the Class IIB subfamily was manually identified and this subset of genes was then used to create a phylogenetic tree.

## Results

### Gene expression analysis across *P. abies* wood revealed expression clusters associated with developmental transitions during wood formation

**Figure 1:**
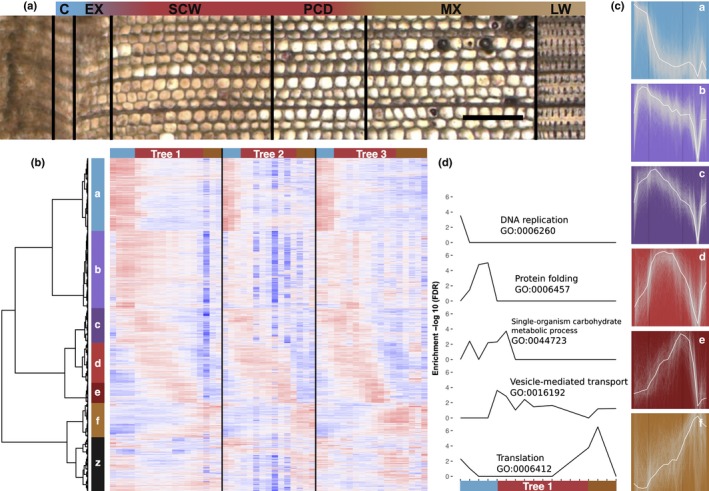
Clustering of *Picea abies* gene expression profiles matching developmental transitions. (a) A representative transverse section stained with nitroblue tetrazolium (NBT) from the stem of Tree 1. A high‐spatial‐resolution gene expression atlas was created by collecting series of tangential cryosections from the stem. The sections were pooled into 14–18 pools in each tree in such a way that representative samples were obtained for each stage of xylem development including cambium (C), xylem expansion (EX), secondary cell wall formation (SCW), programmed cell death (PCD), mature xylem (MX) and the previous year′s latewood formation (LW). Bar, 200 μm. (b) Hierarchical clustering of gene expression data in cryosection series of three trees. Seven gene expression clusters are indicated (a–f, z). Cluster‐z contained genes with no defined profile across the cutting series and was not further analysed. Samples appear in the order of sampling within each tree (T1–T3) and three sample clusters are indicated (blue, red and brown). Expression values are scaled per gene so that expression values above the gene average are represented by red, and below average by blue. (c) Expression profiles of genes within six of the gene clusters. The average expression of each gene cluster is shown in white with all expression profiles from Tree 1 plotted in grey in the background. (d) Genes were assigned to the sample(s) within Tree 1 in which they were expressed within 4% of the maximum expression for that gene, after which gene ontology (GO) enrichment was calculated for each sample. Selected GO categories are represented. All significant categories are available in Supporting Information Table S5.

A high‐spatial‐resolution gene expression data resource was created in *P*. *abies* woody tissues representing developmental stages of cambial through to secondary xylem formation (Fig. 1a; Table S1). In total, 51 samples were assayed for gene expression using deep RNA‐seq profiling, with an average of 15 million aligned paired‐end reads per sample (Table S2). Hierarchical clustering of the gene expression data revealed expression profiles that were highly reproducible among the three replicate trees (Figs 1b, S1), supporting the high quality and reproducibility of the data. The resulting data were integrated into a web resource, ‘NorWood’, to allow community exploration and analysis of gene expression profiles and the associated co‐expression network.

Hierarchical clustering of both genes and samples was performed, which identified seven gene clusters (Fig. 1b,c; Table S3) and three sample clusters (Figs 1b, S2). The gene expression profiles in clusters a–e corresponded well to the different developmental stages of wood formation, with the expression peak of cluster‐a corresponding to cambial tissues, cluster‐b to xylem expansion, cluster‐c to the transition from xylem expansion to SCW formation, cluster‐d to bulk SCW formation, cluster‐e to transition between SCW formation and mature xylem (i.e. programmed cell death) and cluster‐f to mature wood and the previous year's latewood (Fig. 1c). The expression profiles of genes in cluster‐z did not have a distinct developmental profile and this cluster was not considered in subsequent analyses.

GO enrichment analyses of the expression clusters confirmed that they reflected biological processes congruent with the cluster expression domains (Table S4). For example, GO categories related to cell division including ‘regulation of cell cycle’ (GO:0051726) and ‘DNA replication’ (GO:0006260) were enriched in cluster‐a, in agreement with this cluster containing genes having highest expression in close proximity to the dividing cambial cells. Several GO categories related to chromatin status, including ‘chromatin organisation’ (GO:0006325), ‘chromatin modification’ (GO:0016568) and ‘histone modification’ (GO:0016570), were also enriched in cluster‐a. The cellular components ‘cytoskeletal part’ (GO:0044430) and ‘microtubule’ (GO:0005874) were enriched in cluster‐d, and the molecular functions ‘cysteine‐type peptidase activity’ (GO:008233) and ‘hydrolase activity’ (GO:16787) in cluster‐e, supporting the predominance of cell wall formation and cell death in determining the respective cluster expression domains. Similarly, examination of individual genes within the clusters identified numerous candidate genes related to the biological processes occurring within the cluster expression domains (Table S3). For example, cluster‐a included an orthologue of *A. thaliana WOX4*, a known regulator of cambial development (Hirakawa *et al*., 2010). In cluster‐f, terms related to stress and transport were enriched, including ‘response to wounding’ (GO:0009611) and ‘single‐organism transport’ (GO:0044765).

GO enrichment was analysed separately in the different samples in tree T1, which was selected as the replicate with the highest sequencing data and sample quality. To identify the predominant biological processes active within each sample, expression of genes was assigned only to the sample(s) in which a gene was expressed within 4% of its maximal expression. The first sample, T1‐03, was composed entirely of dividing cambial cells and, accordingly, was enriched for GO categories related to cell division, including ‘DNA replication’ (GO:0006260) and ‘protein DNA complex assembly’ (GO:0065004) (Fig. 1d; Table S5). In samples within the Expanding Xylem (EX), terms including ‘cell growth’ (GO:0016049), ‘macromolecule metabolic process’ (GO:0043170), ‘protein folding’ (GO:006457) and ‘carbohydrate metabolic process’ (GO:0005975) were enriched, supporting the predominance of not only cell expansion but also biosynthesis of macromolecules, such as the cell wall carbohydrates, in these samples (Fig. 1d; Table S5). In samples from the SCW zone, categories related to biosynthesis and transport of the SCW components were enriched, as would be expected (Table S5). Notably, the last samples before latewood from the previous year (T1‐17 and 18) had a distinct set of enriched GO terms in comparison to the immediately surrounding samples, which could be related to their unique nature in representing the mature part of the wood, where the only living cell type is the ray cells. Also of note, the last sample (T1‐20), which contained latewood from the previous year, was different from the two immediately preceding samples, suggesting that ray cells may not be the only transcriptionally active cell type in this sample, but that some tracheids could still be living and contribute to the unique gene expression pattern of this sample.

Taken together, the results of both the gene expression clustering and sample enrichment analyses show that coordinated sets of genes display distinct and spatially defined expression profiles, the functional enrichment of which represent biological transitions known to occur along the wood developmental trajectory. Further exploration and validation of this dataset is expected to reveal several novel aspects of xylem differentiation.

### The NorWood web resource

An integrated set of web‐accessible tools was developed as the NorWood (Norway spruce Wood) resource (http://norwood.congenie.org). These tools enable exploration and analysis of the RNA‐seq data resource, with NorWood being integrated within the PlantGenIE (Plant Genome Integrative Explorer; http://plantgenie.org; Sundell *et al*., 2015) web platform to facilitate wider analysis using the extended range of tools and species hosted under the PlantGenIE umbrella. The NorWood web resource can be used to visualise gene expression profiles, explore the pre‐calculated co‐expression network and analyse enrichment of functional categories.

The co‐expression network was used to calculate a number of network centrality measures based on the largest connected sub‐network of 17 942 genes (Table S6). Four measures of network connectivity were calculated and are available at NorWood: degree, betweenness rank, closeness rank and average nearest neighbour degree (see the Materials and Methods section). Degree is used here as the default connectivity measure and represents a score where increasing values indicate an increasing number of connections (edges in the network) to other genes within the network. An underlying assumption is that genes of high degree play a central role within the network and that their removal, or modulation of their expression, would induce extensive knock‐on effects, affecting a large number of other genes. Although correlation provides only circumstantial evidence, and such high‐degree nodes are not necessarily regulators, they are strong candidates for follow‐on biological studies. Similarly, genes with high betweenness can be considered potential switches linking one region of the network to another, for example a gene controlling a developmental transition from cell division to differentiation. Such network metrics can therefore be used to guide biologists to a subset of the most interesting genes for further consideration when starting with (often) considerably long lists of genes identified from other approaches such as differential expression or clustering analyses. Using network metrics can subsequently be combined with other sources of information, such as gene functional annotation, to further filter candidate gene lists. As an example, a gene with the high betweenness rank was MA_10018650g0010, which was located in cluster‐b and is therefore a good candidate regulator for the transition from xylem cell expansion to xylem maturation. This was supported by expression of the closest *A. thaliana* homologue (AT3G15220), a protein kinase of unknown function that is expressed specifically in expanding xylem vessel elements of the primary root within the AtGenExpress dataset (Schmid *et al*., 2005), as visualised in the e‐FP browser (http://bar.utoronto.ca/efp/cgi-bin/efpWeb.cgi; Winter *et al*., 2007) available within the AtGenIE website (http://atgenie.org/efp). Interestingly, the closest neighbour of MA_10018650g0010 within the NorWood expression network is a zinc finger TF (MA_10432012g0010) that is the most sequence‐similar homologue of an *A. thaliana* gene (AT5G05660) predicted to encode the circadian clock component EARLY BIRD (Johansson *et al*., 2011), indicating potential involvement of circadian regulation in the onset of xylem maturation. GO enrichment tests of the top 100 genes ranked by betweeness and centrality did not identify any enriched terms (Table S6).

### Exploiting NorWood integration within PlantGenIE to identify potential *CesA* regulators

**Figure 2:**
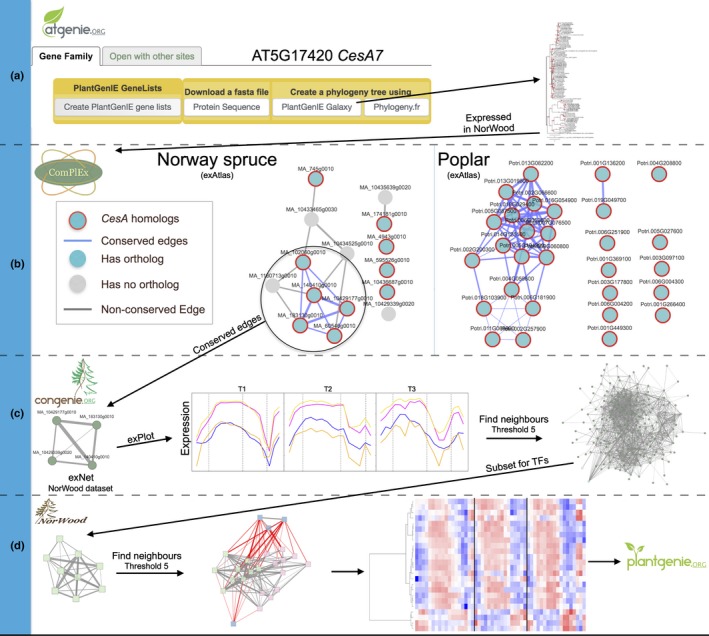
Using the NorWood resource to identify putative regulators of secondary cell wall *cellulose synthase* genes. (a) *Arabidopsis thaliana cellulose synthase A7* (*CesA7*; AT5G17420) was used to find and retrieve the *cellulose synthase* gene family at AtGenIE.org. A phylogenetic tree of the *CesA* gene family for *A. thaliana, Picea abies* (Norway spruce) and *Populus trichocarpa* (poplar) produced using the PlantGenIE phylogenetic tree galaxy workflow is shown. (b) Conservation of co‐expression between the expressed *CesA* gene family members in NorWood and their *P. trichocarpa* orthologues was examined using the complex tool. (c) The network neighbourhood of *CesA* genes with conserved co‐expression was retrieved using the NorWood dataset within the exNet tool at ConGenIE using a context‐likelihood of relatedness (CLR) threshold of 5. This neighbourhood contained four genes comprising MA_183130g0010, MA_140410g0010 and MA_10429177g0010, which are orthologous to the *A. thaliana* secondary cell wall *CesA* genes*,* and one secondary cell wall *CesA*‐like gene, MA_10429339g0020. Shown in this panel on the left is the network, in the centre are line plots from the explot tool with one coloured line representing each of the four neighbourhood genes and the network neighbourhood of these four genes on the right. In the line plots, T1–T3 represent the three replicate trees and dashed vertical lines represent cambium/expanding xylem, secondary cell wall/programmed cell death and mature xylem/late wood zones. (d) The subset of network neighbours annotated as transcription factors (TFs) were selected. The network neighbourhood of this TF subset was then selected at a CLR threshold of 5, subset to genes annotated as TFs, the expression of which were visualised as a heatmap within the NorWood resource. Shown on the left are the starting nine TFs identified in (c), in the centre is the network of TFs where the original nine are shown in green and where neighbours connected by positive edges (i.e. a positive Pearson correlation, indicated as grey edges) are shown in pink and those connected by negative edges (red edges) are shown in blue. In the heatmap expression values are scaled per gene so that expression values above the gene average are represented by red, and below average by blue. Samples appear in the order of sampling within each tree (T1–T3).

To demonstrate the potential of the tools available at NorWood and PlantGenIE, network neighbours were extracted for the *cellulose synthase* (*CesA*) gene family members to identify their potential regulators (Fig. 2). A walk‐through guide to recreating the results presented is available from the NorWood homepage. First, the genelist tool at AtGenIE was used to identify the *A. thaliana CesA* genes (search term ‘cesa’). *CesA7* (AT5G17420) was selected to extract the gene family members in *A. thaliana*,* P*. *trichocarpa* and *P*. *abies* from the Gene Family tab of the *CesA7*‐associated gene information page (Fig. 2a). The phylogenetic tree of these *CesA* genes created using the PlantGenIE Galaxy workflow showed that the *P*. *abies* genome contains putative homologues for primary cell wall (PCW) and SCW *CesA*s, as well as a number of *CesA‐like* (*CSL*) genes (Fig. 2a; Notes S1, section 1.2).

Conservation of the network neighbourhoods was analysed for *P*. *abies CesA* and *CesA‐like* genes that were expressed in NorWood and compared to orthologues in *Populus,* which is the model system for forest tree genomics and also included as a resource in PlantGenIE. Five *P*. *abies* genes had conserved expression with *Populus* (indicated as blue network edges in Fig. 2b). These were passed as a new gene list to ConGenIE and examined in the NorWood dataset using the exNet tool (Fig. 2c), which identified four strongly co‐expressed *CesA* genes. These four genes included one homologue for each of the *A. thaliana CesA* genes *AtCesA4*,* 7* and *8* (Fig. S3; MA_183130g0010, MA_140410g0010, MA_10429177g0010), which are known to control cellulose biosynthesis during SCW formation (McFarlane *et al*., 2014), in addition to one gene (MA_10429339g0020) that was highly similar to the SCW *CesA* genes (see Fig. S3) and that we hereafter refer to as *SCW CesA‐like*. All four were located in cluster‐d, with highest expression during SCW formation. The network neighbourhood of these genes was extracted using the expand function in exNet (at an expansion threshold of 5) after which potential regulators were identified by selecting the subset of TFs (Fig. 2d). This TF subset contained 11 genes (Table 1) including, for example, MA_402393g0010 and MA_95898g0010, which are most sequence‐similar to *A. thaliana ANAC075* (AT4G29230), a known regulator of SCW formation (Endo *et al*., 2014; Sakamoto & Mitsuda, 2015). Subsequently, the TF subset neighbourhood was expanded (threshold 5), which resulted in identification of 21 TFs including the homologue of *A*. *thaliana KNOTTED‐like* (AT4G08150) and a number of *MYB TFs* (Table S7).

**Table 1:** Putative transcription factors identified in the network neighbourhood of *Picea abies* secondary cell wall *cellulose synthase* genes in NorWood

Gene ID	*A*. *thaliana* best BLASTp	Percentage identity	*A*. *thaliana* description
MA_10425867g0020	AT1G69560	52.86	myb domain protein 105
MA_10434782g0020	AT1G65620	66.67	Lateral organ boundaries (LOB) domain family protein
MA_137453g0010	AT3G61250	62.07	myb domain protein 17
MA_139238g0010	AT4G01680	47.76	myb domain protein 55
MA_161608g0010	AT3G27650	68.42	LOB domain‐containing protein 25
MA_30401g0010	AT2G45120	27.23	C2H2‐like zinc finger protein
MA_402393g0010	AT4G29230	40.11	NAC domain containing protein 75
MA_10426405g0010	AT1G02040	35.35	C2H2‐like zinc finger family protein
MA_95898g0010	AT4G29230	61.98	NAC domain containing protein 75

The first‐order neighbours of secondary cell wall *CesA* genes (MA_183130g0010, MA_140410g0010, MA_10429177g0010, which are orthologous to the *Arabidopsis thaliana* secondary cell wall *CesA* genes*,* and one secondary cell wall *CesA*‐like gene, MA_10429339g0020) identified within the NorWood co‐expression network using the exnet tool at ConGenIE.org at a context‐likelihood of relatedness (CLR) threshold of 5. Column headers indicate: Gene ID, *P*. *abies* gene ID as available at http://congenie.org; best *A. thaliana* BLASTp, best *A. thaliana* BLASTp; percentage identity, percentage identity to the best hit *A. thaliana* protein calculated using clustal 2.1; *A. thaliana* description, *Arabidopsis thaliana* gene description from TAIR 10.

We additionally explored the co‐expression and expression domains of the PCW and SCW *CesAs* in ConGenIE.org within the exAtlas and NorWood datasets. Proost & Mutwil (2016) reported that using the ConGenIE exAtlas dataset, it appeared that only one shared co‐expression module was present containing both the PCW and the SCW *CesA* genes (Fig. S4). The greater spatial resolution available in the NorWood dataset resolved the expression domains of the PCW and SCW *CesAs*, revealing two disconnected co‐expression modules. The expression profile plots of the two gene sets clearly showed that SCW *CesAs* were expressed in the SCW forming zone while PCW genes were more ubiquitously expressed across the developmental series, which is an interesting observation suggesting that PCW genes are perhaps more broadly expressed than expected during wood formation in *P. abies*.

### NorWood provides evidence of evolutionarily diverged functions of the NAC Class IIB TFs between angiosperms and gymnosperms

**Figure 3:**
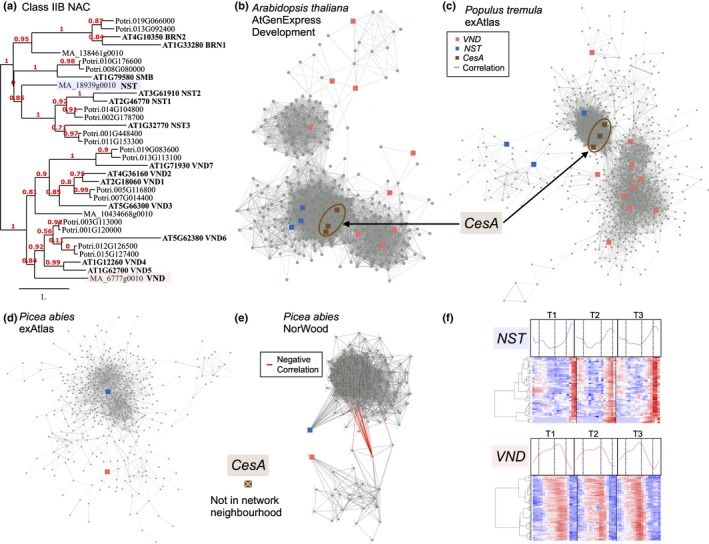
The network neighbourhood of the *Class IIB NAC* family indicates divergence in the regulation of *cellulose synthase* genes. (a) A phylogenetic tree of the Class IIB NAC family. Two of the four *Picea abies* genes were expressed in the NorWood dataset and are highlighted in blue and red. (b) The AtGenExpress Development (Schmid *et al*., 2005) network neighbourhood of the *Arabidopsis thaliana NAC IIB* subfamily was identified using the AtGenIE exnet tool at a context‐likelihood of relatedness (CLR) threshold of 5. Secondary cell wall *cellulose synthase* genes (*CesA4*,* 7* and *8*) are indicated. (c) The *Populus tremula* exAtlas (Sundell *et al*., 2015) network neighbourhood of the *Populus trichocarpa NAC IIB* family was identified using the PopGenIE exnet tool at a CLR threshold of 5. (d) The *P*. *abies* exAtlas (Nystedt *et al*., 2013) network neighbourhood of the *NAC IIB* subfamily was identified using the ConGenIE exnet tool at a CLR threshold of 3.5. A lower CLR threshold was used to ensure a network of comparable size. (e) The NorWood network neighbourhood of the *P. abies NAC IIB* subfamily was identified using the ConGenIE exnet tool at a CLR threshold of 3.5. The *NST* and *VND* orthologues reside in different subnetworks connected with negatively correlated expression profiles (red links). No *CesA* genes were present in the neighbourhood of *NST* or of VND. (f) Gene expression profiles of *P*. *abies VND* and *NST* and heatmap representations of their respective positive network neighbourhood within the NorWood co‐expression network (CLR threshold 5). For the VST and NST homologue, the expression profile is shown for the three replicate trees (T1–T3) where dashed vertical lines represent cambium/expanding xylem, secondary cell wall/programmed cell death and mature xylem/late wood zones. A heatmap of the corresponding network neighbourhood is shown below the expression profile plots with expression values scaled per gene so that expression values above the gene average are represented by red, and below average by blue. Samples appear in the order of sampling within each tree (T1–T3).

Class IIB NAC TFs have been shown to serve as master regulators of the SCW in angiosperms (Nakano *et al*., 2015). The NorWood web resource was used to explore whether there is expression‐based evidence suggesting functional conservation in *P*. *abies*. Nystedt *et al*. (2013) identified four Class IIB family members in the *P*. *abies* genome assembly, of which two (MA_18939g0010 and MA_6777g0010) were highly expressed in the single wood sample of the *P. abies* exAtlas dataset (Nystedt *et al*., 2013; see exImage at ConGenIE.org) as well as in the NorWood dataset. In a phylogenetic tree, MA_18939g0010 clustered together with the *A. thaliana* NST1, NST2 and NST3, and is hereafter called ‘NST’. MA_6777g0010 clustered together with the *A. thaliana* VND4, VND5 and VND6, and is hereafter called ‘VND’ (Fig. 3a). To examine whether the regulation of SCW biosynthesis by the Class IIB NAC TFs is conserved between *A. thaliana, P. tremula* and *P. abies*, the network neighbourhoods of all family members were analysed with the prediction that central SCW biosynthetic genes, such as the SCW *CesAs,* would be present in all three neighbourhoods if regulation is conserved. This prediction was confirmed for *A. thaliana* and *P. tremula* using the exnet tool at AtGenIE (Fig. 3b) and PopGenIE (Fig. 3c), respectively, within the available AtGenExpress (Schmid *et al*., 2005) and *P. tremula* exAtlas (Sundell *et al*., 2015) co‐expression networks, respectively. However, in both the *P. abies* exAtlas (Nystedt *et al*., 2013) and the NorWood datasets, no SCW *CesA* genes were identified in the co‐expression neighbourhoods of either *VND* or *NST*, even when using a lower co‐expression threshold of 3 (Fig. 3d,e).

To identify putative targets for the VND and NST homologues in *P. abies*, their network neighbours were analysed in Norwood, which provides far greater spatial resolution than the *P. abies* exAtlas dataset, which comprises different tissue types (Fig. 3d,e). The network neighbours of *NST* in NorWood included two orthologues of *A. thaliana* lignin biosynthetic Cinnamoyl‐coA reductase1 (MA_137109g0010: AT1G15950; MA_166604g0010: AT1G15950) (Table S8), which is a known target of *A. thaliana* NST1 (Mitsuda *et al*., 2005). The network neighbours of *VND* included several xylem cysteine peptidase genes (MA_10425982g0010: AT4G35350; MA_10429635g0010: AT4G35350; MA_7787614g0010: AT1G20850) (Table S8), which have similarly been identified as targets of *A. thaliana* VND6 and VND7 (Zhong *et al*., 2010; Yamaguchi *et al*., 2011). Even though the *P. abies VND* and *NST* were found in the same network in Norwood, they were connected by negative correlation edges only (Fig. 3e). This was also evident from examination of the expression profiles of *NST* and *VND* (as well as their closest network neighbours) in NorWood: while the expression of the *VND* homologue peaked in the end of the SCW zone, the *NST* homologue was highly expressed throughout the mature xylem (MX) and late wood (LW) zones (Fig. 3f). The differential expression profiles and network neighbourhoods strongly suggest separate regulatory roles for the two Class IIB NAC TFs in *P*. *abies*.

## Discussion

High‐spatial‐resolution RNA‐seq gene expression profiles were generated from replicated, tangential cryo‐section series spanning the wood development zone in *P*. *abies* and deposited to an interactive, web‐based tool – NorWood. While such large‐scale RNA‐seq data resources have an inherent value, their true potential only becomes evident and accessible to the wider community when they are made available in an intuitive, visual and interactive form. In addition, evo‐devo insights require such data to be integrated within platforms that enable direct, comparative analyses of cross‐species co‐expression networks to facilitate identification of conserved and diverged regulatory mechanisms and co‐expression profiles. While a number of excellent web resources are available, many have a focus on coding sequence‐based comparisons. For example, the PLAZA (Proost *et al*., 2015) and Phytozome (Goodstein *et al*., 2012) resources provide extensive coverage of both angiosperm and gymnosperm (in the case of PLAZA) species, with cross‐species gene family information allowing exploration of protein coding gene conservation. Similarly, a number of resources integrate gene expression and protein–protein interaction data, including PlaNet, PODC, CORNET and ATTED‐II (Mutwil *et al*., 2011; De Bodt *et al*., 2012; Ohyanagi *et al*., 2015; Aoki *et al*., 2016). However, the majority of these are focused towards agricultural food crop species and *A. thaliana*. This limits their ability to provide insights into biological processes that are not well represented by, or are lacking in, these species, such as wood formation, seasonal dormancy and senescence or perennial growth. PlantGenIE (Sundell *et al*., 2015), by contrast, has a focus on tree species and seamlessly integrates sequence and expression information to investigate the conservation of co‐expression networks, which represents a powerful means to uncover evolutionary divergence driven by underlying changes in gene co‐expression modules. Here, the power of developing a gene expression resource profiling wood development across the annual growth ring of *P*. *abies* is demonstrated, revealing how the high spatial resolution available from sampling mature trees enables detailed insights into the expression domain of genes regulating the process of wood formation.

As conifers are long‐lived and have to cope with changing environmental conditions, previous global gene expression analyses have often focused on resistance or acclimation responses or mechanisms (e.g. Verne *et al*., 2011; Yeaman *et al*., 2014). There have also been a number of transcriptome analyses of xylem tissues in various conifer species (e.g. Pavy *et al*., 2008; Li *et al*., 2011; Raherison *et al*., 2015), largely motivated by the ecological and economical value of wood. However, as the samples in these previous studies originated from multiple wood cell layers, it has been difficult to identify genes that are expressed during specific phases of tracheid development. In the current work, the spatial information on these phases was preserved in the form of RNA‐seq data generated from tangential cryosections taken throughout an annual growth ring, which represented all transitions during tracheid development. A novel characteristic of the current data is that it includes samples from mature xylem and the previous year's latewood, in which most of the cells (i.e. tracheids) are thought to be dead, with the exception of ray cells (Nakaba *et al*., 2008). Therefore, the transcriptomes of samples within the MX and LW zones are likely to be derived from those living rays cells only. In support of this, genes within cluster‐f showed high expression specifically in the MX and LW zones and were enriched for GO terms congruent with the biological processes active within ray cells. Such insights have not previously been possible using low‐resolution sampling, where mature wood was either omitted entirely or where the transcriptome of the mature part of the wood would have been combined with that of additional developmental zones within the developing xylem.

In angiosperms, recent studies have begun to resolve the transcriptional network controlling SCW formation, and NAC domain proteins, a plant‐specific TF family, have been shown to play a central role in this process (e.g. Nakano *et al*., 2015; Taylor‐Teeples *et al*., 2015). In conifers there is evidence that aspects of these regulatory mechanisms may differ: Rigault *et al*. (2011) investigated the distribution of protein domains between different species and showed that the NAC domain is one of the most underrepresented in *P. glauca* compared to studied angiosperm species (Rigault *et al*., 2011). In line with this, *P*. *abies* has only four genes encoding members of the Class IIB subfamily of NAC domain proteins in contrast to 13 genes in *A*. *thaliana* (Fig. 3a and Nystedt *et al*., 2013). The expression profiles and co‐expression network neighbourhoods of the two Class IIB family genes expressed in the NorWood data were examined and found to include a single *NST* and a single *VND* homologue (Fig. 3). Surprisingly, the genes had distinctly contrasting expression profiles in *P*. *abies* wood; the expression of the *VND* gene peaked in the SCW zone whereas the *NST* gene was most highly expressed much later, in the MX and LW zones (Fig. 3f). When the co‐expression network neighbourhoods of the *P. abies NST* and *VND* were examined in NorWood, no SCW *CesA* genes were found, whereas co‐expression networks in both *A. thaliana* and *P. tremula* identified SCW *CesA* genes as neighbours of *NST1* (Fig. 3b–e). Taken together, these results indicate a divergence in the regulation of SCW formation between the two angiosperm species and *P*. *abies* in addition to demonstrating the importance of high‐spatial‐resolution transcriptional data to resolve expression domains.

To provide a worked example, the NorWood resource was used to identify a set of candidate TFs representing potentially novel regulators of SCW *CesA* genes in conifers and to demonstrate how the integrated resources within PlantGenIE can be utilised to perform comparative regulomics analyses to explore their evolutionary conservation. Based on phylogenetic analysis and examination of expression patterns, three bona fide *P*. *abies* SCW *CesA* genes were identified (Figs 2a, S3), in agreement with previous studies performed in other coniferous species (Nairn & Haselkorn, 2005; Raherison *et al*., 2012). Interestingly, the expression of two *P. abies ANAC075* (AT4G29230) homologues correlated with expression of the SCW *CesA* genes in NorWood. Even though ANAC075 has not been directly shown to regulate expression of SCW CesAs, it has been demonstrated to induce ectopic differentiation of xylem vessel elements, accompanied by SCW deposition, when overexpressed in *A. thaliana* (Endo *et al*., 2014). Also, SND2, which is the closest homologue of ANAC075 in *A. thaliana*, is known to stimulate SCW formation in fibres (Hussey *et al*., 2011) and directly regulate expression of *CesA8* (Zhong *et al*., 2008). These data support functional conservation of the NAC factors, such as ANAC075 and SND2, between angiosperms and gymnosperms while the Class IIB NAC factors seem to have gained central function in regulation of SCW biosynthesis only after divergence of the angiosperms and gymnosperms. Furthermore, the fact that the moss *Physcomitrella patens* possesses Class IIB NAC TFs that are functionally conserved between *P. patens* and *A. thaliana* (Xu *et al*., 2014) supports a view that the Class IIB NAC TFs obtained new functionalities in the angiosperms through changes in the gene expression domain rather than the protein function. Further high‐spatial co‐expression analyses as well as functional assays of these genes across the kingdoms are needed to confirm the evolutionary history of SCW regulation.

Here, use of the NorWood web resource is exemplified to provide novel evo‐devo insights into the divergent regulation of developmental process during wood formation between *P. abies,* as a representative conifer, and the angiosperm model species *A. thaliana* and *Populus* species. The resource represents a powerful tool for hypotheses generation for subsequent validation studies and for advancing understanding of wood formation in coniferous species in addition to enabling evo‐devo focused analyses. In specific cases, such comparative genomics approaches can hold particular value where candidate genes are essential, and therefore intractable to knock‐out approaches, for example. More importantly, they enable discovery of novel candidates that would not be considered given current understanding.

## Author contributions

S.J‐L. performed sample collection and preparation and contributed to manuscript writing and interpretation of the results. D.S. performed the analysis of the RNA‐seq data, designed and implemented the NorWood resource and contributed to writing the manuscript. O.N. contributed to the experimental design and data interpretation. T.R.H. performed comparative regulomics analyses, contributed to the design of the NorWood resource, interpretation of the results and manuscript writing. N.R.S. contributed to data interpretation and manuscript writing. H.T. conceived and implemented the study and contributed to manuscript writing.

## Supporting information

Please note: Wiley Blackwell are not responsible for the content or functionality of any Supporting Information supplied by the authors. Any queries (other than missing material) should be directed to the *New Phytologist* Central Office.

**Fig. S1** Principal component analysis plot of normalised expression values.
**Fig. S2** Gene expression heatmap indicating sample and gene clustering.
**Fig. S3** Midpoint rooted phylogenetic tree of *Picea abies*,* Arabidopsis thaliana* and *Populus trichocarpa cellulose synthase* (*CesA*) and *CesA‐like* (*CSL*) subfamilies B, D, E and G.
**Fig. S4** Co‐expression among primary and secondary cell wall *cellulose synthase* genes.
**Notes S1** Supplementary figures, genotype confirmation for biological replicates, phylogenetic analysis and gene model annotation refinement for *cellulose synthase* family members.

**Table S1** Section pooling and cell profiling

**Table S2** Gene expression and sequence data quality

**Table S3** Hierarchical clustering of genes

**Table S4** Gene ontology enrichment of hierarchical clusters

**Table S5** Gene ontology enrichment per sample

**Table S6** Network statistics

**Table S7** Transcription factor network neighbours of the secondary cell wall *cellulose synthase* genes in *Picea abies*

**Table S8** Secondary cell wall *cellulose synthase A* gene network neighbourhoods

## References

[nph14458-bib-0001] Aoki Y , Okamura Y , Tadaka S , Kinoshita K , Obayashi T . 2016 ATTED‐II in 2016: a plant coexpression database towards lineage‐specific coexpression. Plant and Cell Physiology 57: e5. 26546318 10.1093/pcp/pcv165 PMC4722172

[nph14458-bib-0002] Barabási A‐L , Oltvai ZN . 2004 Network biology: understanding the cell's functional organization. Nature Reviews Genetics 5: 101–113. 10.1038/nrg1272 14735121

[nph14458-bib-0003] Bedon F , Grima‐Pettenati J , MacKay J . 2007 Conifer R2R3‐MYB transcription factors: sequence analyses and gene expression in wood‐forming tissues of white spruce (*Picea glauca*). BMC Plant Biology 7: 17. 17397551 10.1186/1471-2229-7-17 PMC1851958

[nph14458-bib-0004] Berlyn GP , Miksche JP . 1976 Botanical microtechnique and cytochemistry. Ames, IA, USA: Iowa State University Press.

[nph14458-bib-0005] Bolger AM , Lohse M , Usadel B . 2014 Trimmomatic: a flexible trimmer for Illumina sequence data. Bioinformatics 30: 2114–2120. 24695404 10.1093/bioinformatics/btu170 PMC4103590

[nph14458-bib-0006] Bomal C , Bedon F , Caron S , Mansfield SD , Levasseur C , Cooke JE , Blais S , Tremblay L , Morency MJ , Pavy N *et al* 2008 Involvement of *Pinus taeda MYB1* and *MYB8* in phenylpropanoid metabolism and secondary cell wall biogenesis: a comparative *in planta* analysis. Journal of Experimental Botany 59: 3925–3939. 18805909 10.1093/jxb/ern234 PMC2576632

[nph14458-bib-0007] Courtois‐Moreau CL , Pesquet E , Sjödin A , Muñiz L , Bollhöner B , Kaneda M , Samuels L , Jansson S , Tuominen H . 2009 A unique program for cell death in xylem fibers of *Populus* stem. Plant Journal 58: 260–274. 19175765 10.1111/j.1365-313X.2008.03777.x

[nph14458-bib-0008] Daub CO , Steuer R , Selbig J , Kloska S . 2004 Estimating mutual information using B‐spline functions – an improved similarity measure for analysing gene expression data. BMC Bioinformatics 5: 118. 15339346 10.1186/1471-2105-5-118 PMC516800

[nph14458-bib-0009] De Bodt S , Hollunder J , Nelissen H , Meulemeester N , Inzé D . 2012 CORNET 2.0: integrating plant coexpression, protein‐protein interactions, regulatory interactions, gene associations and functional annotations. New Phytologist 195: 707–720. 22651224 10.1111/j.1469-8137.2012.04184.x

[nph14458-bib-0010] De La Torre AR , Birol I , Bousquet J , Ingvarsson PK , Jansson S , Jones SJM , Keeling CI , MacKay J , Nilsson O , Ritland K *et al* 2014 Insights into conifer giga‐genomes. Plant Physiology 166: 1724–1732. 25349325 10.1104/pp.114.248708 PMC4256843

[nph14458-bib-0011] Delhomme N , Mähler N , Schiffthaler B , Sundell D , Mannapperuma C , Hvidsten TR , Street NR . 2014 Guidelines for RNA‐seq data analysis. *Epigenesys Protocols (prot 67)* [WWW document] URL http://www.epigenesys.eu/en/protocols/bio-informatics/1283-guidelines-for-rna-seq-data-analysis [accessed 24 June 2014].

[nph14458-bib-0012] Dobin A , Davis CA , Schlesinger F , Drenkow J , Zaleski C , Jha S , Batut P , Chaisson M , Gingeras TR . 2013 STAR: ultrafast universal RNA‐seq aligner. Bioinformatics 29: 15–21. 23104886 10.1093/bioinformatics/bts635 PMC3530905

[nph14458-bib-0013] Duval I , Lachance D , Giguère I , Bomal C , Morency MJ , Pelletier G , Boyle B , MacKay JJ , Séguin A . 2014 Large‐scale screening of transcription factor–promoter interactions in spruce reveals a transcriptional network involved in vascular development. Journal of Experimental Botany 65: 2319–2333. 24713992 10.1093/jxb/eru116 PMC4036505

[nph14458-bib-0014] Edgar RC . 2004 MUSCLE: multiple sequence alignment with high accuracy and high throughput. Nucleic Acids Research 32: 1792–1797. 15034147 10.1093/nar/gkh340 PMC390337

[nph14458-bib-0015] Endo H , Yamaguchi M , Tamura T , Nakano Y , Nishikubo N , Yoneda A , Kato K , Kubo M , Kajita S , Katayama Y *et al* 2014 Multiple classes of transcription factors regulate the expression of *VASCULAR‐RELATED NAC‐DOMAIN7*, a master switch of xylem vessel differentiation. Plant and Cell Physiology 56: 242–254. 25265867 10.1093/pcp/pcu134

[nph14458-bib-0016] Faith JJ , Hayete B , Thaden JT , Mogno I , Wierzbowski J , Cottarel G , Kasif S , Collins JJ , Gardner TS . 2007 Large‐scale mapping and validation of *Escherichia coli* transcriptional regulation from a compendium of expression profiles. PLoS Biology 5: e8. 17214507 10.1371/journal.pbio.0050008 PMC1764438

[nph14458-bib-0017] Gahan PB . 1984 Plant histochemistry and cytochemistry. London, UK: Academic Press.

[nph14458-bib-0018] Goecks J , Nekrutenko A , Taylor J , The Galaxy Team . 2010 Galaxy: a comprehensive approach for supporting accessible, reproducible, and transparent computational research in the life sciences. Genome Biology 11: R86. 20738864 10.1186/gb-2010-11-8-r86 PMC2945788

[nph14458-bib-0019] Goodstein DM , Shu S , Howson R , Neupane R , Hayes RD , Fazo J , Mitros T , Dirks W , Hellsten U , Putnam N *et al* 2012 Phytozome: a comparative platform for green plant genomics. Nucleic Acids Research 40: D1178–D1186. 22110026 10.1093/nar/gkr944 PMC3245001

[nph14458-bib-0021] Guindon S , Dufayard JF , Lefort V , Anisimova M , Hordijk W , Gascuel O . 2010 New algorithms and methods to estimate maximum‐likelihood phylogenies: assessing the performance of PhyML 3.0. Systematic Biology 59: 307–321. 20525638 10.1093/sysbio/syq010

[nph14458-bib-0022] Guo M , Song W , Buhain J . 2015 Bioenergy and biofuels: history, status, and perspective. Renewable and Sustainable Energy Reviews 42: 712–725.

[nph14458-bib-0023] Havimo M , Rikola J , Sirviö J , Sipi M . 2008 Distribution of tracheid cross‐sectional dimensions in different parts of Norway spruce stems. Silva Fennica 42: 89–99.

[nph14458-bib-0024] Hertzberg M , Aspeborg H , Schrader J , Andersson A , Erlandsson R , Blomqvist K , Bhalerao R , Uhlen M , Teeri TT , Lundeberg J *et al* 2001 A transcriptional roadmap to wood formation. Proceedings of the National Academy of Sciences, USA 98: 14732–14737. 10.1073/pnas.261293398 PMC64750 11724959

[nph14458-bib-0025] Hirakawa Y , Kondo Y , Fukuda H . 2010 TDIF peptide signaling regulates vascular stem cell proliferation via the *WOX4* homeobox gene in *Arabidopsis* . Plant Cell 22: 2618–2629. 20729381 10.1105/tpc.110.076083 PMC2947162

[nph14458-bib-0026] Hussey SG , Mizrachi E , Creux NM , Myburg AA . 2013 Navigating the transcriptional roadmap regulating plant secondary cell wall deposition. Frontiers in Plant Science 4: 325. 24009617 10.3389/fpls.2013.00325 PMC3756741

[nph14458-bib-0027] Hussey SG , Mizrachi E , Spokevicius AV , Bossinger G , Berger DK , Myburg AA . 2011 *SND2*, a NAC transcription factor gene, regulates genes involved in secondary cell wall development in *Arabidopsis* fibres and increases fibre cell area in *Eucalyptus* . BMC Plant Biology 11: 173. 22133261 10.1186/1471-2229-11-173 PMC3289092

[nph14458-bib-0028] Immanen J , Nieminen K , Smolander O‐P , Kojima M , Alonso Serra J , Koskinen P , Zhang J , Elo A , Mähönen AP , Street N *et al* 2016 Cytokinin and auxin display distinct but interconnected distribution and signaling profiles to stimulate cambial activity. Current Biology 26: 1990–1997. 27426519 10.1016/j.cub.2016.05.053

[nph14458-bib-0029] Isikgor FH , Becer CR . 2015 Lignocellulosic biomass: a sustainable platform for the production of bio‐based chemicals and polymers. Polymer Chemistry 6: 4497–4559.

[nph14458-bib-0030] Jin J , Tian F , Yang D‐C , Meng Y‐Q , Kong L , Luo J , Gao G . 2016 PlantTFDB 4.0: toward a central hub for transcription factors and regulatory interactions in plants. Nucleic Acids Research 42: 2856–2869. 10.1093/nar/gkw982 PMC5210657 27924042

[nph14458-bib-0031] Johansson M , McWatters HG , Bakó L , Takata N , Gyula P , Hall A , Somers DE , Millar AJ , Eriksson ME . 2011 Partners in time: EARLY BIRD associates with ZEITLUPE and regulates the speed of the Arabidopsis clock. Plant Physiology 155: 2108–2122. 21300918 10.1104/pp.110.167155 PMC3091123

[nph14458-bib-0032] Kubo M , Udagawa M , Nishikubo N , Horiguchi G , Yamaguchi M , Ito J , Mimura T , Fukuda H , Demura T . 2005 Transcription switches for protoxylem and metaxylem vessel formation. Genes & Development 19: 1855–1860. 16103214 10.1101/gad.1331305 PMC1186185

[nph14458-bib-0033] Li X , Wu HX , Southerton SG . 2010 Seasonal reorganization of the xylem transcriptome at different tree ages reveals novel insights into wood formation in *Pinus radiata* . New Phytologist 187: 764–776. 20561208 10.1111/j.1469-8137.2010.03333.x

[nph14458-bib-0034] Li XG , Wu HX , Southerton SG . 2011 Transcriptome profiling of *Pinus radiata* juvenile wood with contrasting stiffness identifies putative candidate genes involved in microfibril orientation and cell wall mechanics. BMC Genomics 12: 480. 21962175 10.1186/1471-2164-12-480 PMC3224210

[nph14458-bib-0035] Lopes CT , Franz M , Kazi F , Donaldson SL , Morris Q , Bader GD . 2010 Cytoscape Web: an interactive web‐based network browser. Bioinformatics 26: 2347–2348. 20656902 10.1093/bioinformatics/btq430 PMC2935447

[nph14458-bib-0036] Love MI , Huber W , Anders S . 2014 Moderated estimation of fold change and dispersion for RNA‐seq data with DESeq2. Genome Biology 15: 550. 25516281 10.1186/s13059-014-0550-8 PMC4302049

[nph14458-bib-0037] MacNeil LT , Walhout AJM . 2011 Gene regulatory networks and the role of robustness and stochasticity in the control of gene expression. Genome Research 21: 645–657. 21324878 10.1101/gr.097378.109 PMC3083081

[nph14458-bib-0038] McFarlane HE , Döring A , Persson S . 2014 The cell biology of cellulose synthesis. Annual Review of Plant Biology 65: 69–94. 10.1146/annurev-arplant-050213-040240 24579997

[nph14458-bib-0039] Mitsuda N , Iwase A , Yamamoto H , Yoshida M , Seki M , Shinozaki K , Ohme‐Takagi M . 2007 NAC transcription factors, NST1 and NST3, are key regulators of the formation of secondary walls in woody tissues of *Arabidopsis* . Plant Cell 19: 270–280. 17237351 10.1105/tpc.106.047043 PMC1820955

[nph14458-bib-0040] Mitsuda N , Seki M , Shinozaki K , Ohme‐Takagi M . 2005 The NAC transcription factors NST1 and NST2 of *Arabidopsis* regulate secondary wall thickenings and are required for anther dehiscence. Plant Cell 17: 2993–3006. 16214898 10.1105/tpc.105.036004 PMC1276025

[nph14458-bib-0041] Moreau C , Aksenov N , Lorenzo M , Segerman B , Funk C , Nilsson P , Jansson S , Tuominen H . 2005 A genomic approach to investigate developmental cell death in woody tissues of *Populus* trees. Genome Biology 6: R34. 15833121 10.1186/gb-2005-6-4-r34 PMC1088962

[nph14458-bib-0042] Mutwil M , Klie S , Tohge T , Giorgi FM , Wilkins O , Campbell MM , Fernie AR , Usadel B , Nikoloski Z , Persson S . 2011 PlaNet: combined sequence and expression comparisons across plant networks derived from seven species. Plant Cell 23: 895–910. 21441431 10.1105/tpc.111.083667 PMC3082271

[nph14458-bib-0043] Nairn CJ , Haselkorn T . 2005 Three loblolly pine *CesA* genes expressed in developing xylem are orthologous to secondary cell wall *CesA* genes of angiosperms. New Phytologist 166: 907–915. 15869651 10.1111/j.1469-8137.2005.01372.x

[nph14458-bib-0044] Nakaba S , Kubo T , Funada R . 2008 Differences in patterns of cell death between ray parenchyma cells and ray tracheids in the conifers *Pinus densiflora* and *Pinus rigida* . Trees 22: 623–630.

[nph14458-bib-0045] Nakano Y , Yamaguchi M , Endo H , Rejab NA , Ohtani M . 2015 NAC‐MYB‐based transcriptional regulation of secondary cell wall biosynthesis in land plants. Frontiers in Plant Science 6: 288. 25999964 10.3389/fpls.2015.00288 PMC4419676

[nph14458-bib-0046] Netotea S , Sundell D , Street NR , Hvidsten TR . 2014 ComPlEx: conservation and divergence of co‐expression networks in *A*. *thaliana*,* Populus* and *O*. *sativa* . BMC Genomics 15: 106. 24498971 10.1186/1471-2164-15-106 PMC3925997

[nph14458-bib-0047] Nystedt B , Street NR , Wetterbom A , Zuccolo A , Lin Y‐C , Scofield DG , Vezzi F , Delhomme N , Giacomello S , Alexeyenko A *et al* 2013 The Norway spruce genome sequence and conifer genome evolution. Nature 497: 579–584. 23698360 10.1038/nature12211

[nph14458-bib-0048] Ohyanagi H , Takano T , Terashima S , Kobayashi M , Kanno M , Morimoto K , Kanege H , Sasaki Y , Saito M , Asano S *et al* 2015 Plant omics data center: an integrated web repository for interspecies gene expression networks with NLP‐based curation. Plant and Cell Physiology 56: e9. 25505034 10.1093/pcp/pcu188 PMC4301748

[nph14458-bib-0049] Paiva JAP , Garnier‐Géré PH , Rodrigues JC , Alves A , Santos S , Graça J , Le Provost G , Chaumeil P , Da Silva‐Perez D , Bosc A *et al* 2008 Plasticity of maritime pine (*Pinus pinaster*) wood‐forming tissues during a growing season. New Phytologist 179: 1080–1094. 18631295 10.1111/j.1469-8137.2008.02536.x

[nph14458-bib-0050] Pavy N , Boyle B , Nelson C , Paule C , Giguère I , Caron S , Parsons LS , Dallaire N , Bedon F , Bérubé H *et al* 2008 Identification of conserved core xylem gene sets: conifer cDNA microarray development, transcript profiling and computational analyses. New Phytologist 180: 766–786. 18811621 10.1111/j.1469-8137.2008.02615.x

[nph14458-bib-0051] Pethica R , Barker G , Kovacs T , Gough J . 2010 TreeVector: scalable, interactive, phylogenetic trees for the web. PLoS ONE 5: e8934. 20126613 10.1371/journal.pone.0008934 PMC2812488

[nph14458-bib-0052] Proost S , Mutwil M . 2016 Tools of the trade: studying molecular networks in plants. Current Opinion in Plant Biology 30: 143–150. 26990519 10.1016/j.pbi.2016.02.010

[nph14458-bib-0053] Proost S , Van Bel M , Veneechoutte D , Van de Peer Y , Inzé D , Mueller‐Roeber B , Vandepoele K . 2015 PLAZA 3.0: an access point for plant comparative genomics. Nucleic Acids Research 43: D974–D981. 25324309 10.1093/nar/gku986 PMC4384038

[nph14458-bib-0054] Raherison ESM , Giguère I , Caron S , Lamara M , MacKay JJ . 2015 Modular organization of the white spruce (*Picea glauca*) transcriptome reveals functional organization and evolutionary signatures. New Phytologist 207: 172–187. 25728802 10.1111/nph.13343 PMC5024012

[nph14458-bib-0055] Raherison E , Rigault P , Caron S , Poulin P‐L , Boyle B , Verta J‐P , Giguère I , Bomal C , Bohlmann J , MacKay J . 2012 Transcriptome profiling in conifers and the PiceaGenExpress database show patterns of diversification within gene families and interspecific conservation in vascular gene expression. BMC Genomics 13: 434. 22931377 10.1186/1471-2164-13-434 PMC3534630

[nph14458-bib-0056] Rigault P , Boyle B , Lepage P , Cooke JEK , Bousquet J , MacKay JJ . 2011 A white spruce gene catalog for conifer genome analyses. Plant Physiology 157: 14–28. 21730200 10.1104/pp.111.179663 PMC3165865

[nph14458-bib-0057] Sakamoto S , Mitsuda N . 2015 Reconstitution of a secondary cell wall in a secondary cell wall‐deficient Arabidopsis mutant. Plant and Cell Physiology 56: 299–310. 25535195 10.1093/pcp/pcu208 PMC4323883

[nph14458-bib-0058] Schmid M , Davison TS , Henz SR , Pape UJ , Demar M , Vingron M , Schölkopf B , Weigel D , Lohmann JU . 2005 A gene expression map of *Arabidopsis thaliana* development. Nature Genetics 37: 501–506. 15806101 10.1038/ng1543

[nph14458-bib-0059] Schrader J , Nilsson J , Mellerowicz E , Berglund A , Nilsson P , Hertzberg M , Sandberg G . 2004 A high‐resolution transcript profile across the wood‐forming meristem of poplar identifies potential regulators of cambial stem cell identity. Plant Cell 16: 2278–2292. 15316113 10.1105/tpc.104.024190 PMC520933

[nph14458-bib-0060] Stephenson PG , Harris N , Cottrell JE , Ralph SG , Bohlmann J , Taylor G . 2011 A transcriptomic approach to identify genes associated with wood density in *Picea sitchensis* . Scandinavian Journal of Forest Research 26: 82–96.

[nph14458-bib-0061] Sundell D , Mannapperuma C , Netotea S , Delhomme N , Lin Y‐C , Sjödin A , Van de Peer Y , Jansson S , Hvidsten TR . 2015 The Plant Genome Integrative Explorer Resource: PlantGenIE.org. New Phytologist 208: 1149–1156. 26192091 10.1111/nph.13557

[nph14458-bib-0062] Taylor‐Teeples M , Lin L , de Lucas M , Turco G , Toal TW , Gaudinier A , Young NF , Trabucco GM , Veling MT , Lamothe R *et al* 2015 An *Arabidopsis* gene regulatory network for secondary cell wall synthesis. Nature 517: 571–575. 25533953 10.1038/nature14099 PMC4333722

[nph14458-bib-0063] Uggla C , Moritz T , Sandberg G , Sundberg B . 1996 Auxin as a positional signal in pattern formation in plants. Proceedings of the National Academy of Sciences, USA 93: 9282–9286. 10.1073/pnas.93.17.9282 PMC38633 11607701

[nph14458-bib-0064] Verne S , Jaquish B , White R , Ritland C , Ritland K . 2011 Global transcriptome analysis of constitutive resistance to the white pine weevil in spruce. Genome Biology and Evolution 3: 851–867. 21852250 10.1093/gbe/evr069 PMC3296464

[nph14458-bib-0065] Villalobos DP , Díaz‐Moreno SM , Said E‐SS , Cañas RA , Osuna D , Van Kerckhoven SHE , Bautista R , Gonzalo Claros M , Cánovas FM , Cantón FR . 2012 Reprogramming of gene expression during compression wood formation in pine: coordinated modulation of S‐adenosylmethionine, lignin and lignin related genes. BMC Plant Biology 12: 100. 22747794 10.1186/1471-2229-12-100 PMC3406974

[nph14458-bib-0066] Wang X‐Q , Ran J‐H . 2014 Evolution and biogeography of gymnosperms. Molecular Phylogenetics and Evolution 75: 24–40. 24565948 10.1016/j.ympev.2014.02.005

[nph14458-bib-0067] Warnes GR , Bolker B , Bonebakker L , Gentleman R , Huber W , Liaw A , Lumley T , Maechler M , Magnusson A , Moeller S *et al* 2016 gplots: Various R programming tools for plotting data. R package v.3.0.1. [WWW document] URL https://CRAN.R-project.org/package=gplots [accessed 24 June 2014].

[nph14458-bib-0068] Winter D , Vinegar B , Nahal H , Ammar R , Wilson GV , Provart NJ . 2007 An ‘Electronic Fluorescent Pictograph’ browser for exploring and analyzing large‐scale biological data sets. PLoS ONE 2: e718. 17684564 10.1371/journal.pone.0000718 PMC1934936

[nph14458-bib-0069] Xu B , Ohtani M , Yamaguchi M , Toyooka K , Wakazaki M , Sato M , Kubo M , Nakano Y , Sano R , Hiwatashi Y *et al* 2014 Contribution of NAC transcription factors to plant adaptation to land. Science 343: 1505–1508. 24652936 10.1126/science.1248417

[nph14458-bib-0070] Yamaguchi M , Kubo M , Fukuda H , Demura T . 2008 VASCULAR‐RELATED NAC‐DOMAIN7 is involved in the differentiation of all types of xylem vessels in Arabidopsis roots and shoots. Plant Journal 55: 652–664. 18445131 10.1111/j.1365-313X.2008.03533.x

[nph14458-bib-0071] Yamaguchi M , Mitsuda N , Ohtani M , Ohme‐Takagi M , Kato K , Demura T . 2011 VASCULAR‐RELATED NAC‐DOMAIN 7 directly regulates the expression of a broad range of genes for xylem vessel formation. Plant Journal 66: 579–590. 21284754 10.1111/j.1365-313X.2011.04514.x

[nph14458-bib-0072] Yeaman S , Hodgins KA , Suren H , Nurkowski KA , Rieseberg LH , Holliday JA , Aitken SN . 2014 Conservation and divergence of gene expression plasticity following c. 140 million years of evolution in lodgepole pine (*Pinus contorta*) and interior spruce (*Picea glauca* × *Picea engelmannii*). New Phytologist 203: 578–591. 24750196 10.1111/nph.12819

[nph14458-bib-0073] Zhong R , Lee C , Ye Z‐H . 2010 Global analysis of direct targets of secondary wall NAC master switches in *Arabidopsis* . Molecular Plant 3: 1087–1103. 20935069 10.1093/mp/ssq062

[nph14458-bib-0074] Zhong R , Lee C , Zhou J , McCarthy RL , Ye Z‐H . 2008 A battery of transcription factors involved in the regulation of secondary cell wall biosynthesis in *Arabidopsis* . Plant Cell 20: 2763–2782. 18952777 10.1105/tpc.108.061325 PMC2590737

[nph14458-bib-0075] Zhong R , Richardson EA , Ye ZH . 2007 Two NAC domain transcription factors, SND1 and NST1, function redundantly in regulation of secondary wall synthesis in fibers of *Arabidopsis* . Planta 225: 1603–1611. 17333250 10.1007/s00425-007-0498-y

