# Supplementary material for: NorWood: a gene expression resource for evo‐devo studies of conifer wood development
Source: New Phytol. 2017 Feb 10;216(2):482–94. doi: 10.1111/nph.14458 (PMC6079643; doi:10.1111/nph.14458)
Supplement: Supplementary file 1 — Fig. S1 Principal component analysis plot of normalised expression values. Fig. S2 Gene expression heatmap indicating sample and gene clustering. Fig. S3 Midpoint rooted phylogenetic tree of Picea abies, Arabidopsis thaliana and Populus trichocarpa cellulose synthase (CesA) and CesA‐like (CSL) subfamilies B, D, E and G. Fig. S4 Co‐expression among primary and secondary cell wall cellulose synthase genes. Notes S1 Supplementary figures, genotype confirmation for biological replicates, phylogenetic analysis and gene model annotation refinement for cellulose synthase family members. [file NPH-216-482-s001.pdf]

New Phytologist Supporting Information

Article title: **NorWood: a gene expression resource for evo-devo studies of conifer wood development**

Authors: Soile Jokipii-Lukkari, David Sundell, Ove Nilsson, Torgeir R. Hvidsten, Nathaniel R. Street and Hannele Tuominen

Article acceptance date: 22 December 2016

The following Supporting Information is available for this article:

Fig. S1 Principal component analysis plot of normalised expression values.
Fig. S2 Gene expression heatmap indicating sample and gene clustering.
Fig. S3 Midpoint rooted phylogenetic tree of *Picea abies*, *Arabidopsis thaliana* and *Populus trichocarpa cellulose synthase* (*CesA*) and *CesA-like* (*CSL*) subfamilies B, D, E and G.
Fig. S4 Co-expression among primary and secondary cell wall *cellulose synthase* genes.

Notes S1 Supplementary figures, genotype confirmation for biological replicates, phylogenetic analysis and gene model annotation refinement for *cellulose synthase* family members.

Table S1 Section pooling and cell profiling (see separate file).
Table S2 Gene expression and sequence data quality (see separate file).
Table S3 Hierarchical clustering of genes (see separate file).
Table S4 Gene ontology enrichment of hierarchical clusters (see separate file).
Table S5 Gene ontology enrichment per sample (see separate file).
Table S6 Network statistics (see separate file).
Table S7 Transcription factor network neighbours of the secondary cell wall *cellulose synthase* genes in *Picea abies* (see separate file).
Table S8 Secondary cell wall *cellulose synthase A* gene network neighbourhoods (see separate file).

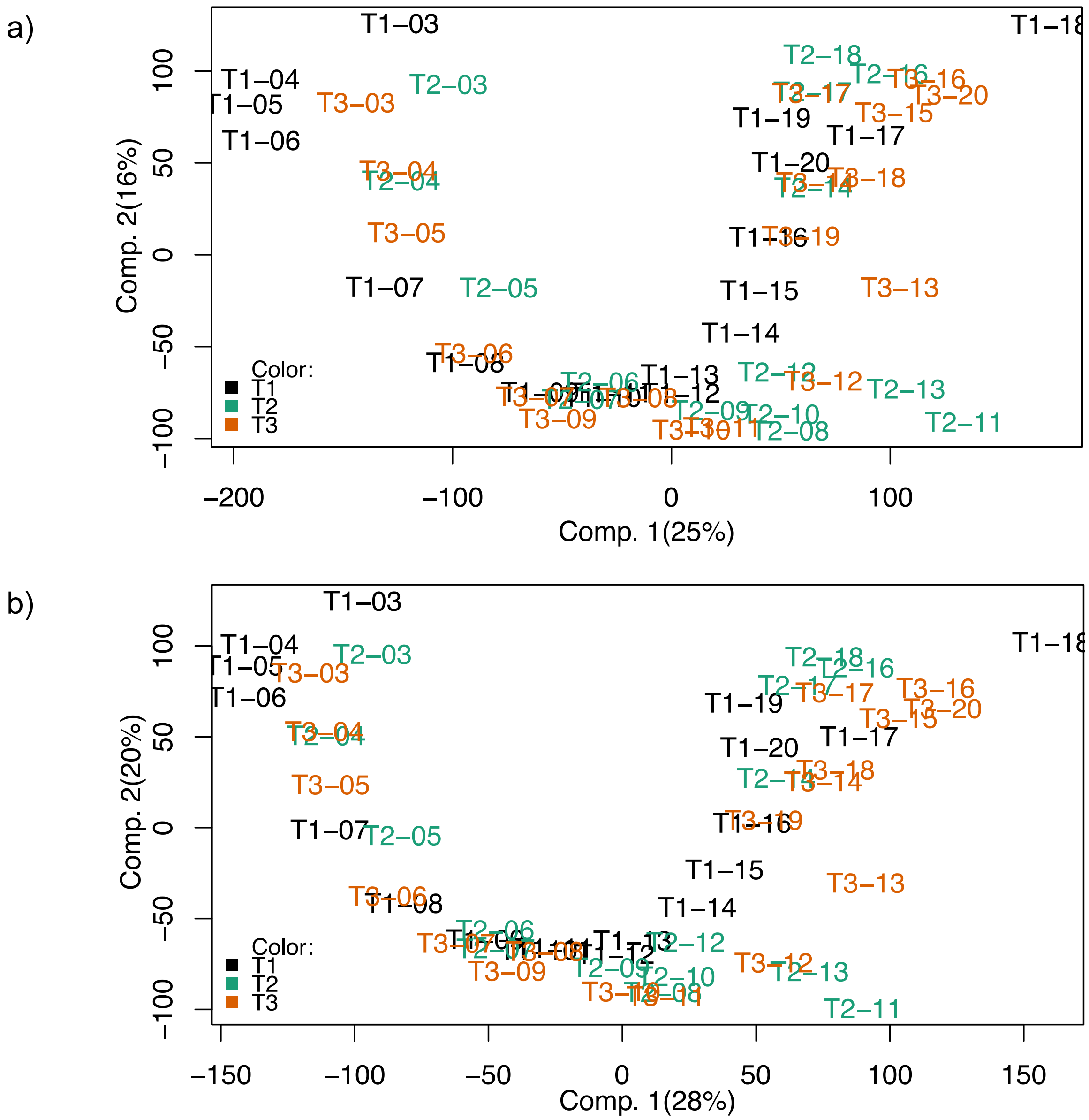

Fig. S1 Principal component analysis plot of normalised expression values. (**a**) The first two principal components of the Variance Stabilised Transformation (VST) normalised expression data. (**b**) The first two principal components after an expression filter was applied requiring a gene to be expressed in at least two samples in two of the three replicates.

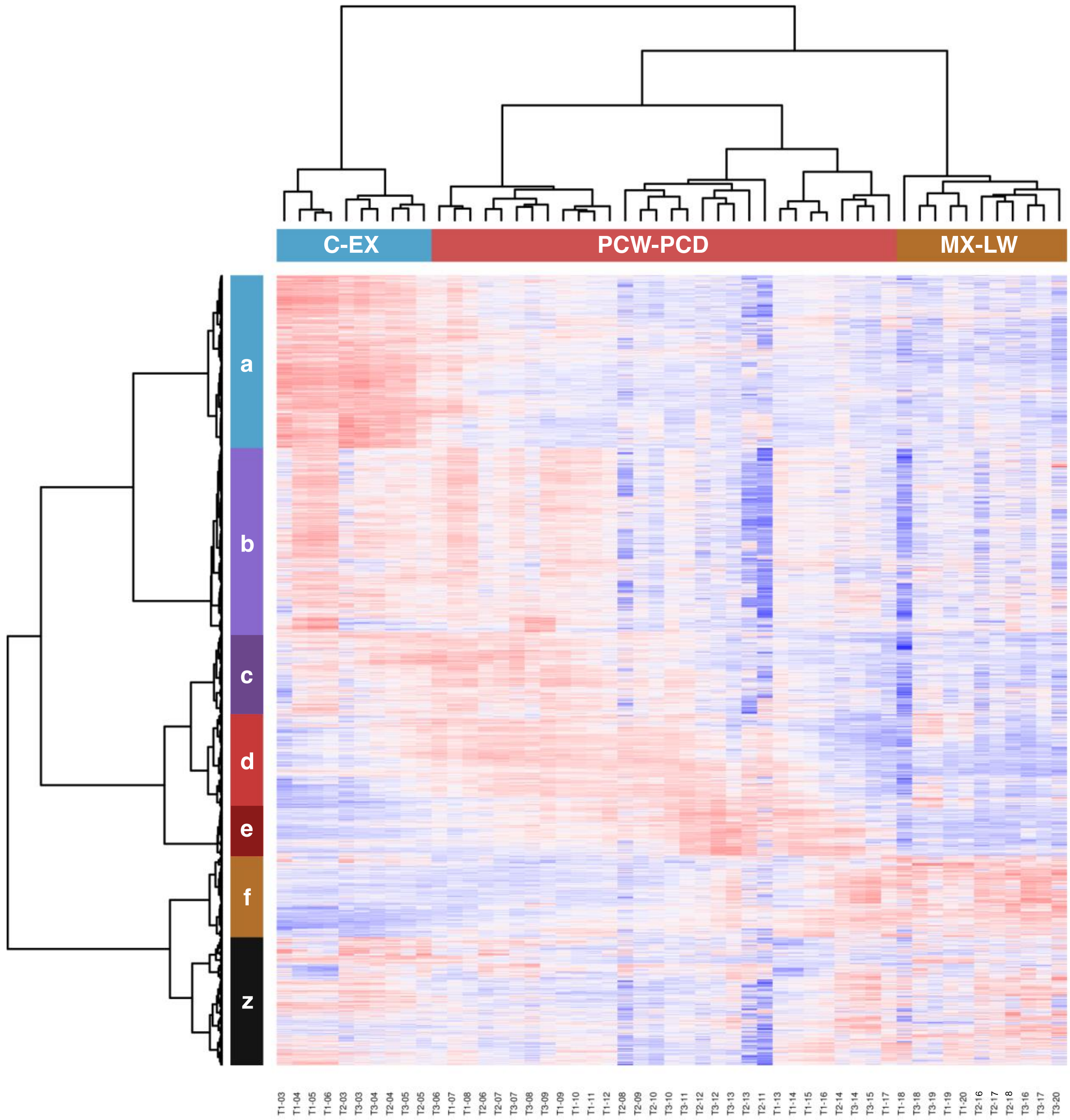

Fig. S2 Gene expression heatmap indicating sample and gene clustering. A heatmap after hierarchical clustering of samples and genes. The samples are divided into three clusters Cambium/Expanding xylem (C/EX; Blue), Secondary Cell Wall/Programmed Cell Death (SCW/PCD; Red), and Mature Xylem/Late Wood (MX/LW; Brown). The clustered genes are divided into seven clusters a (Blue), b (Light purple), c (Dark purple), d (Light red), e (Dark red), f (Brown) and z (Black). Expression values are scaled per gene so that expression values above the gene average are represented by red, and below average by blue.

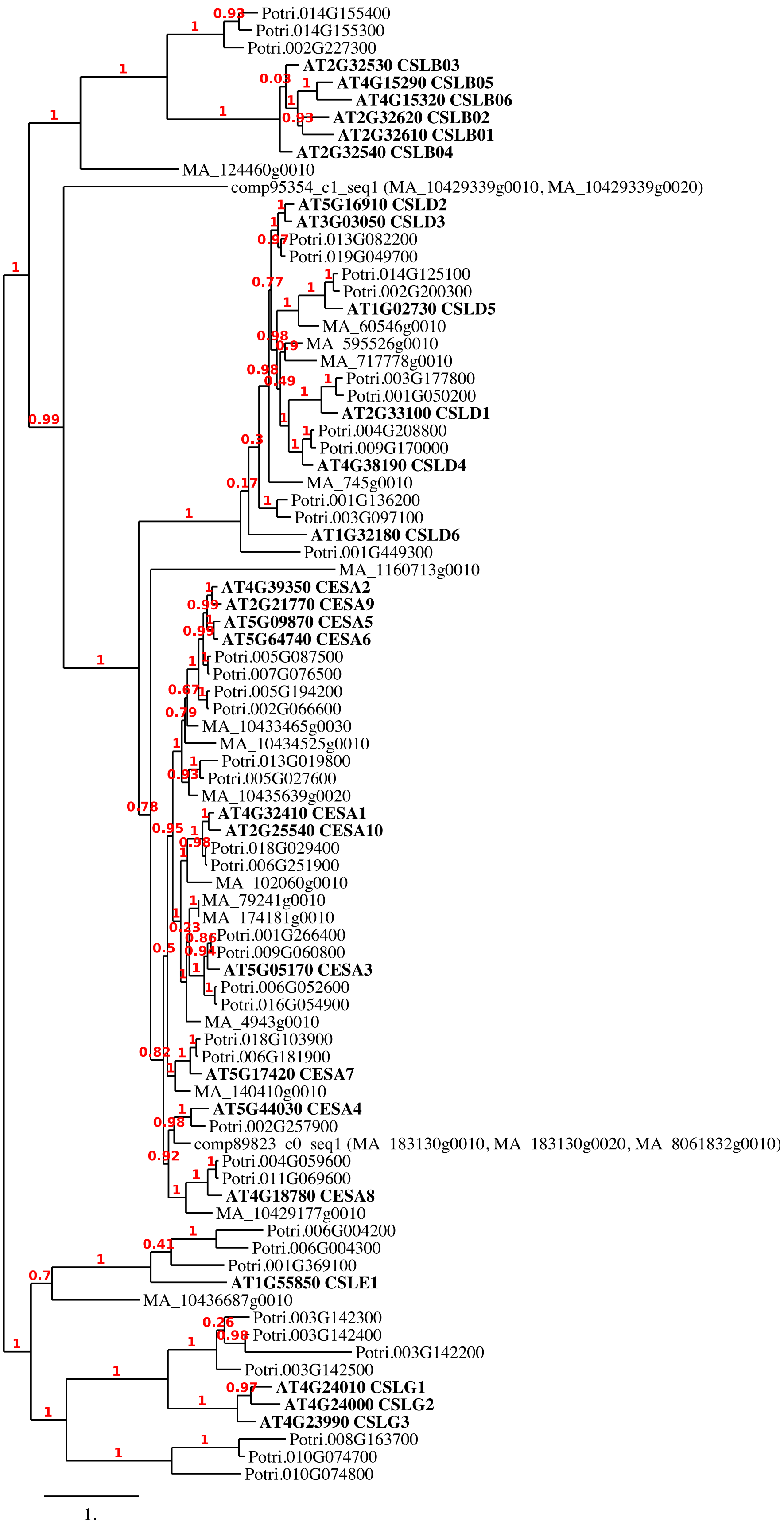

Fig. S3 Midpoint rooted phylogenetic tree of *Picea abies*, *Arabidopsis thaliana* and *Populus trichocarpa cellulose synthase* (*CesA*) and *CesA-like* (*CSL*) subfamilies B, D, E and G. The *A. thaliana* genes are in bold. The values indicate the branch support given by the approximate likelihood ratio test.

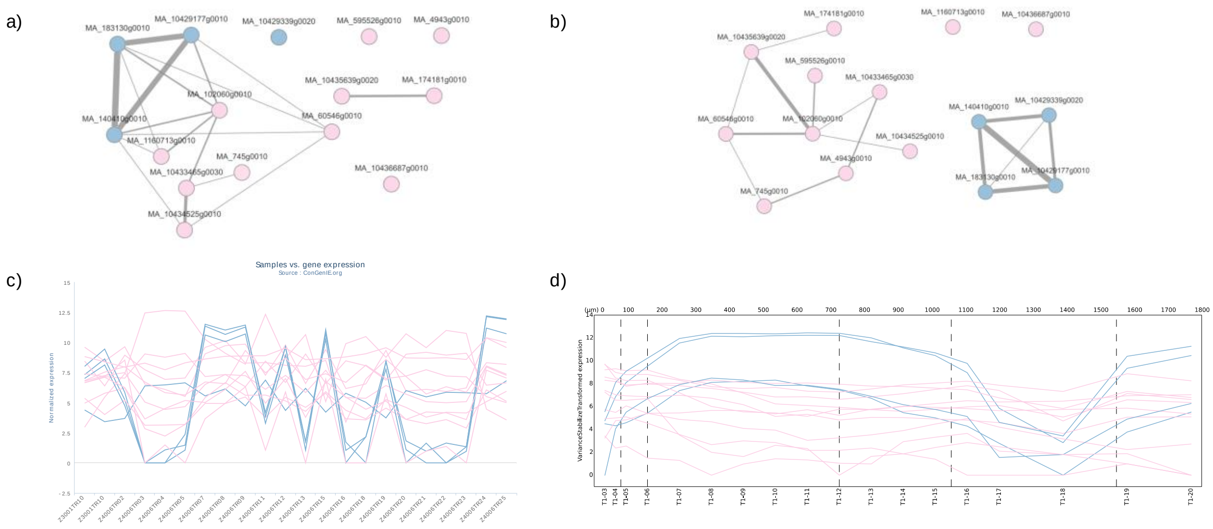

Fig. S4 Co-expression among primary and secondary cell wall *cellulose synthase* genes. Primary cell wall (PCW) *cellulose synthase* (*CesA*) genes are shown in pink and secondary cell wall (SCW) genes in blue. (**a**) Co-expression among the PCW and SCW *CesA* genes in the ConGenIE.org expression Atlas (exAtlas) dataset as identified using the exNet tool with a co-expression threshold of three. (**b**) Co-expression among the PCW and SCW *CesA* genes in the NorWood dataset, as identified using the exNet tool with a co-expression threshold of three. (**c**) Expression profiles of the PCW and SCW *CesA* genes across the exAtlas samples. The plot is generated using the exPlot tool at ConGenIE.org. The y axis shows normalised/scaled expression values (variance-stabilizing transformation RNA-Seq). (**d**) Expression profiles of the PCW and SCW *CesA* genes across the NorWood samples. The plot is generated using the NorWood resource.

Notes S1

1.1 Genotyping

To confirm that the three trees were clonal biological replicates of genotype ‘Z4006’ a genotype test was performed looking at Single Nucleotide Polymorphisms (SNPs) in genes from the top 100 longest scaffolds in the genome assembly (Nystedt *et al.*, 2013). Read information from the three replicates, as well as three samples from an independent experiment used here as a control, were merged using samtools-1.3.1 mpileup (-d100000). SNPs were called using bcftools-1.3.1 call (-v -c). A Principle Component Analysis (PCA) plot was created from the resulting variant call format (vcf; Li, 2011) file using the R-3.3.0 and the package SNPRelate-1.6.4 (Zheng *et al.*, 2012). From the PCA plot (Fig. A) it was clear that the replicates from the NorWood tree sample series formed a tight cluster in contrast to the three control samples, which were distinctly different to the NorWood samples as well as being variable among themselves. This pattern was expected as the control samples were not obtained from clonal replicates.

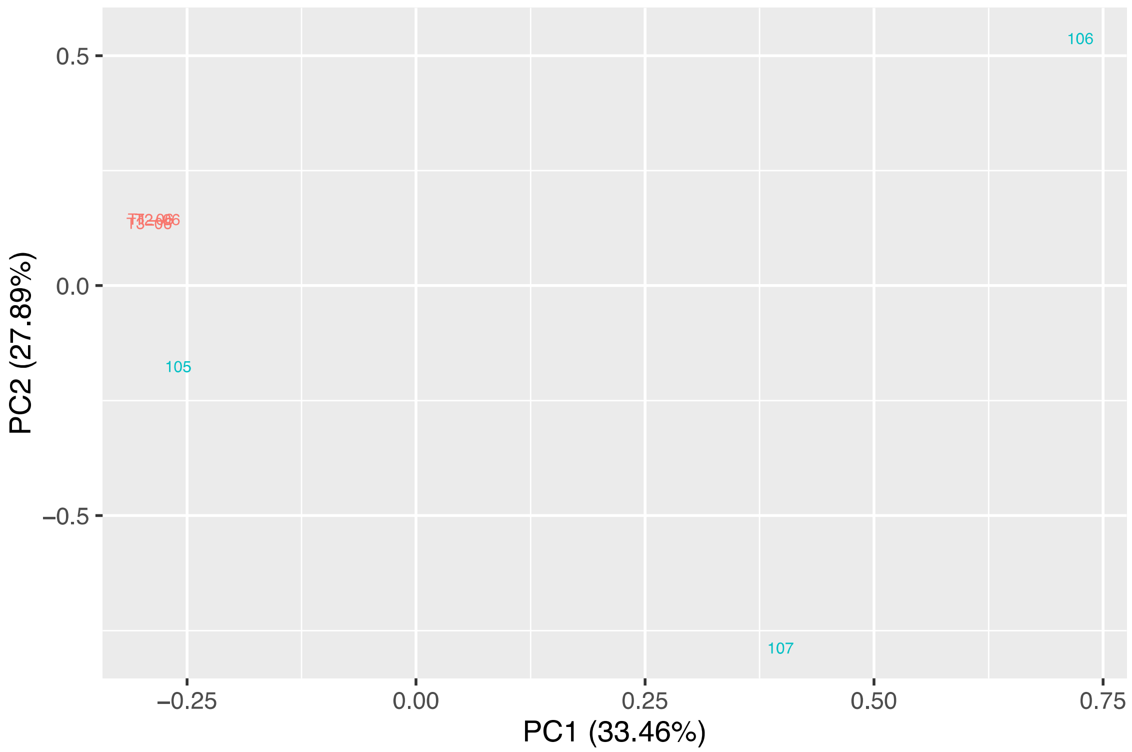

Fig. A. Genotyping of clone Z4006. A PCA-plot of SNPs calls from genes on the 100 longest scaffolds shows that the three clones (red) forms a tight cluster while the three independent control samples (blue) distinctly separates from the three clones.

1.2 Merging genes

An initial phylogenetic tree (Fig. B) of all genes in the cellulose synthase gene family (as identified in PlantGenIE). Two clusters of genes (highlighted in blue in Fig. B below) contained examples of fragmented gene annotation, with two gene fragments located adjacent to each other on a single scaffold in the genome assembly. In both cases evidence was found supporting a merge of the gene models. Samtools-1.3.1 (Li, 2011) mpileup (-d1600 -C50 -d1600) was used to merge reads from several samples (T[1-3]-08 to T[1-3]-12) in the region of both genes (MA_10429339:200-6,500), spanning MA_10429339g0010 (466 - 2979) and MA_10429339g0020 (3047-5917), from which a consensus sequence was called using bcftools-1.3.1 call (-c) and vcfutils.pl vcf2fq (which is included with bcftools). In addition the GBrowse genome browser at ConGenIE.org showed an existing assembled transcript (Trinity RNA-Seq assembly; comp95354_c1_seq1), as shown in Fig. C, spanning both regions. A multiple-sequence alignment produced using MUSCLE (v3.8.31; Edgar, 2004; McWilliam *et al.*, 2013) showed that the consensus sequence and the read sequence were almost identical (Fig. Ca). For the final phylogenetic tree (Fig. S3), a new gene model was predicted from transcript comp95354_c1_seq1 using EMBOSS Transeq (Rice *et al.*, 2000; McWilliam *et al.*, 2013). The resultant gene model covered both of the original, but fragmented, gene models (Figs Ca, D).

The method was repeated on scaffold MA_183130 in the region MA_183130:3000-13000 and supported a merge of the two gene models MA_183130g0010 (3629-11651 bp) and

MA_183130g0020 (11711-12567 bp). The scaffold region also contained an aligned transcript (comp89823_c0_seq1; Fig. Cb). A MUSCLE multiple alignment including the third gene model (MA_ 8061832g0010) located in the cluster together with the other two models indicated in Fig. B showed that MA_8061832g0010 was a fragment with 100% identity to the merged gene model on comp_89823_c0_seq1 (Fig. E).

The newly derived gene models were added to the current version of the genome using WebApollo (Lee *et al*., 2013; Sundell *et al.*, 2015) at ConGenIE.org.

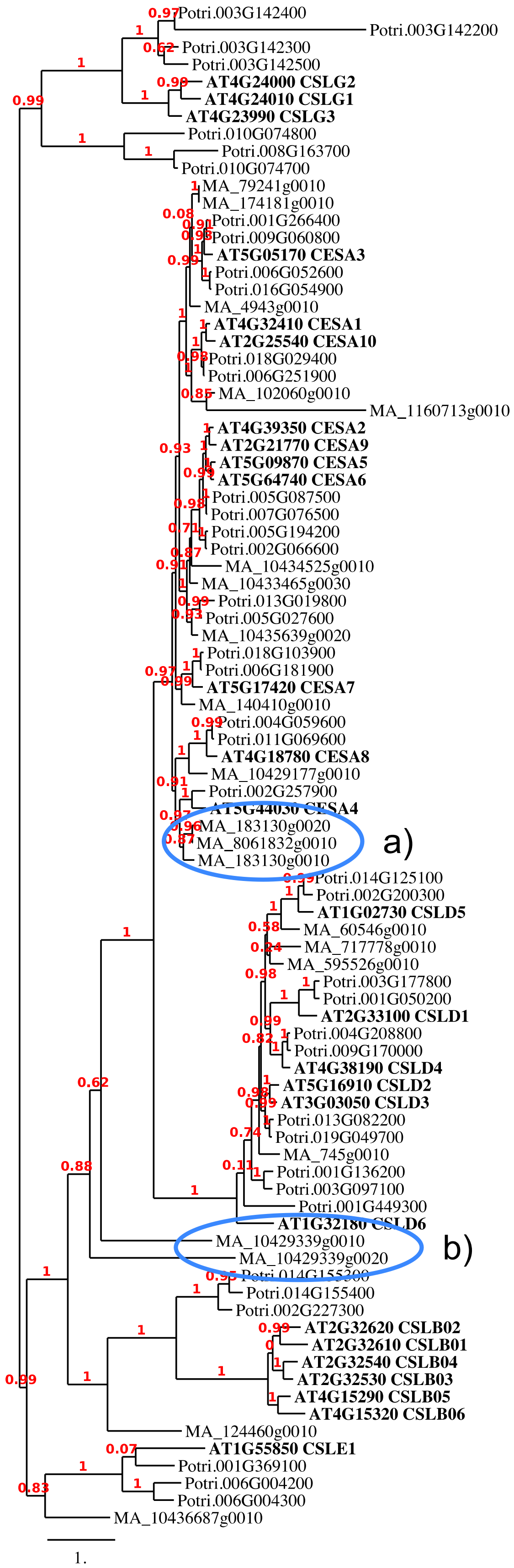

Fig. B. Phylogenetic tree of the cellulose synthase A gene family. The phylogenetic tree shows two clusters (labelled a and b) highlighted with blue ovals that each contain cases of fragmented gene annotations located on the same genome assembly scaffold.

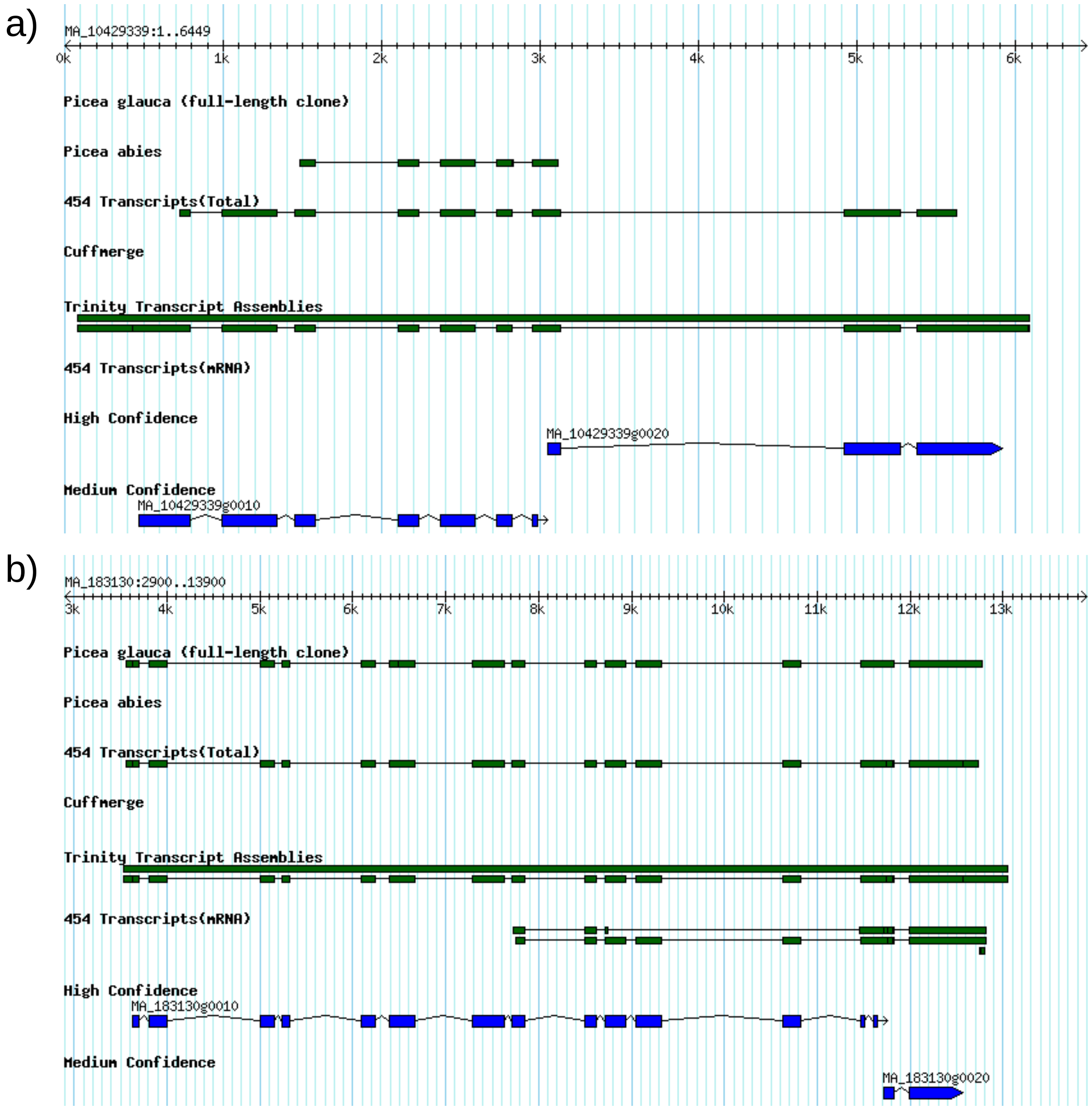

Fig. C. GBrowse view of merged genes including source tracks for the *Picea abies* assembly and *Picea glauca* gene models. GBrowse view of (**a**) MA_10429339 and (**b**) MA_183130, including the annotation tracks *Picea glauca*, *Picea abies*, 454 transcripts (Total), Cuffmerge, Trinity Transcript Assemblies 454 transcripts mRNA, which all represent EST or RNA-Sequencing alignments, and the annotated High- and Medium-Confidence gene models.

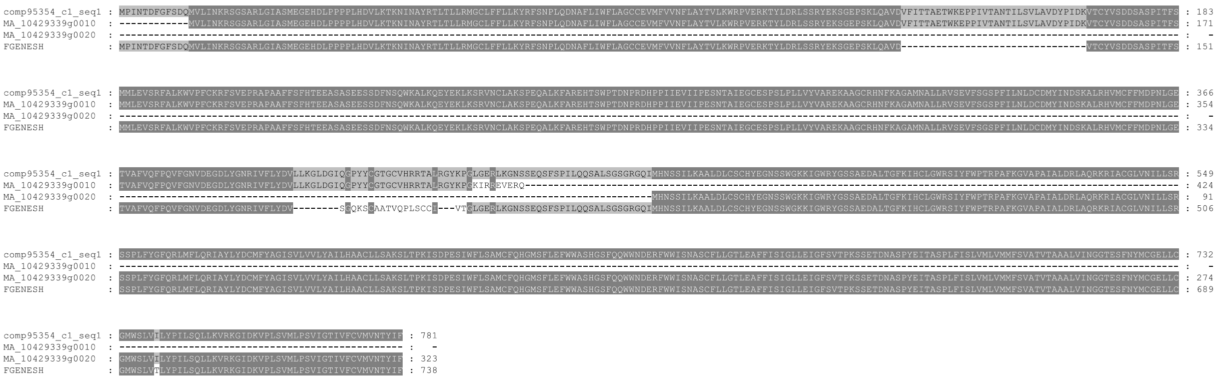

Fig. D MA_10429339. MUSCLE multiple alignment of an EMBOSS Transeq gene prediction of comp95354_c1_seq1, the two gene models marked (b) in Fig. 1 (MA_10429339g0010 and MA_10429339g0020) and a gene prediction of the consensus sequence produced using FgenesH.

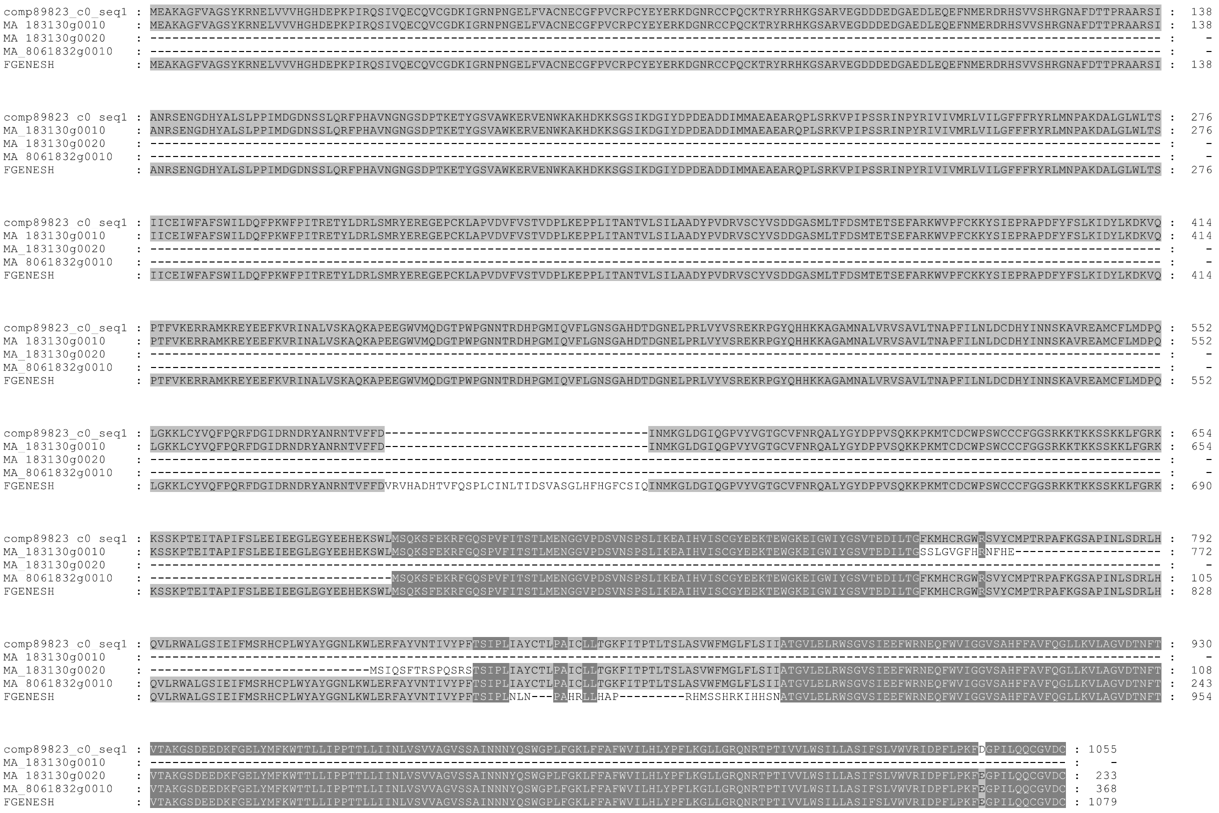

Fig. E MA_183130. MUSCLE multiple alignment of comp89823_c0_seq1, the three gene models labelled (a) in Fig. 1 (MA_183130g0010, MA_183130g0020, MA_8061832g0010) and a gene prediction of the consensus sequence produced using FgenesH.

References

Edgar RC. **2004**. MUSCLE: multiple sequence alignment with high accuracy and high throughput. *Nucleic Acids Research* **32**: 1792–1797.

Lee E, Helt GA, Reese JT, Munoz-Torres MC, Childers CP, Buels RM, Stein L, Holmes IH, Elsik CG, Lewis SE. **2013**. Web Apollo: a web-based genomic annotation editing platform. *Genome Biology* **14**: R93.

Li H. **2011**. A statistical framework for SNP calling, mutation discovery, association mapping and population genetical parameter estimation from sequencing data. *Bioinformatics* **27**: 2987–2993.

McWilliam H, Li W, Uludag M, Squizzato S, Park YM, Buso N, Cowley AP, Lopez R. **2013**. Analysis Tool Web Services from the EMBL-EBI. *Nucleic Acids Research* **41**: 597–600.

Nystedt B, Street NR, Wetterbom A, Zuccolo A, Lin Y-C, Scofield DG, Vezzi F, Delhomme N, Giacomello S, Alexeyenko A *et al.* 2013. The Norway spruce genome sequence and conifer genome evolution. *Nature* **497**: 579–584.

Rice P. **2000**. EMBOSS: The European Molecular Biology Open Software Suite. *Trends in Genetics* **16**: 276–277.

Sundell D, Mannapperuma C, Netotea S, Delhomme N, Lin Y-C, Sjödin A, Van de Peer Y, Jansson S, Hvidsten TR, Street NR. **2015**. The Plant Genome Integrative Explorer Resource: PlantGenIE.org. *New Phytologist* **208**: 1149–1156.

Zheng X, Levine D, Shen J, Gogarten SM, Laurie C, Weir BS. **2012**. A high-performance computing toolset for relatedness and principal component analysis of SNP data. *Bioinformatics* **28**: 3326–3328.
